# Fiber-Reinforced Composites Used in the Manufacture of Marine Decks: A Review

**DOI:** 10.3390/polym17172345

**Published:** 2025-08-29

**Authors:** Lahiru Wijewickrama, Janitha Jeewantha, G. Indika P. Perera, Omar Alajarmeh, Jayantha Epaarachchi

**Affiliations:** 1 Department of Mechanical and Automotive Engineering, Faculty of Engineering & Technology, CINEC Campus, Malabe 10120, Sri Lanka; lahiru.wijewickrama@cinec.edu (L.W.); indika.perera@cinec.edu (G.I.P.P.); 2 Centre for Future Materials, Institute for Advanced Engineering and Space Sciences, University of Southern Queensland, Toowoomba, QLD 4350, Australia; janitha.jeewantha@unisq.edu.au (J.J.); omar.alajarmeh@unisq.edu.au (O.A.); 3 School of Engineering, Faculty of Health Engineering and Sciences, University of Southern Queensland, Toowoomba, QLD 4350, Australia; 4 Department of Mechanical and Manufacturing Engineering, Faculty of Engineering, University of Ruhuna, Matara 81000, Sri Lanka; 5 Department of Civil and Environmental Engineering, United Arab Emirates University, Al Ain 15551, United Arab Emirates

**Keywords:** fiber-reinforced composites, marine structures, durability, manufacturing processes, sustainable materials

## Abstract

Fiber-reinforced composites (FRCs) have emerged as transformative alternatives to traditional marine construction materials, owing to their superior corrosion resistance, design flexibility, and strength-to-weight ratio. This review comprehensively examines the current state of FRC technologies in marine deck and underwater applications, with a focus on manufacturing methods, durability challenges, and future innovations. Thermoset polymer composites, particularly those with epoxy and vinyl ester matrices, continue to dominate marine applications due to their mechanical robustness and processing maturity. In contrast, thermoplastic composites such as Polyether Ether Ketone (PEEK) and Polyether Ketone Ketone (PEKK) offer advantages in recyclability and hydrothermal performance but are hindered by higher processing costs. The review evaluates the performance of various fiber types, including glass, carbon, basalt, and aramid, highlighting the trade-offs between cost, mechanical properties, and environmental resistance. Manufacturing processes such as vacuum-assisted resin transfer molding (VARTM) and automated fiber placement (AFP) enable efficient production but face limitations in scalability and in-field repair. Key durability concerns include seawater-induced degradation, moisture absorption, interfacial debonding, galvanic corrosion in FRP–metal hybrids, and biofouling. The paper also explores emerging strategies such as self-healing polymers, nano-enhanced coatings, and hybrid fiber architectures that aim to improve long-term reliability. Finally, it outlines future research directions, including the development of smart composites with embedded structural health monitoring (SHM), bio-based resin systems, and standardized certification protocols to support broader industry adoption. This review aims to guide ongoing research and development efforts toward more sustainable, high-performance marine composite systems.

## 1. Introduction

### 1.1. Overview of Marine Deck Applications and Their Structural Demands

Marine decks are among the most crucial structural components in the design and function of ships, submarines, offshore platforms, and deep-sea installations. These structures are subjected to extremely harsh environmental conditions that include saltwater-induced corrosion, wave and current-driven hydrodynamic forces, biofouling from marine organisms, and continuous cyclic loading. These factors necessitate the use of materials that can maintain mechanical performance and structural integrity over prolonged periods [1,2]. Furthermore, the high-performance requirements of marine decks include a superior strength-to-weight ratio, fatigue resistance, and minimal degradation over time. Traditionally, metallic materials such as steel and aluminum have dominated this space due to their favorable strength-to-weight characteristics and well-established structural familiarity. However, their inherent disadvantages under marine conditions have prompted researchers and engineers to seek alternatives that can withstand long-term environmental exposure without compromising durability and function [3,4]. The increasing demands for fuel-efficient vessels and durable offshore infrastructure have made lightweight and corrosion-resistant materials not just advantageous but essential. As the marine industry moves toward more sustainable and cost-effective solutions, understanding the structural requirements of decks and how new materials can meet these demands is becoming more critical than ever [1,5].

### 1.2. Conventional Materials and Their Limitations in Marine Environments

Steel and aluminum, while traditionally reliable for marine construction, exhibit substantial drawbacks in seawater environments. Steel, although strong and widely available, is highly susceptible to electrochemical corrosion when exposed to saltwater. This degradation requires the frequent application of protective coatings, cathodic protection, and costly maintenance procedures throughout the lifecycle of marine structures [6,7]. Additionally, the heavyweight of steel increases the dead load of marine vessels and offshore installations, which results in greater fuel consumption and limitations on design flexibility [2]. Aluminum offers a lighter alternative and is favored in many naval and high-speed vessel applications. However, its lower stiffness compared to steel and its vulnerability to galvanic corrosion, especially when in contact with other metals, limit its effectiveness in complex or deep-sea environments [1,8].

Furthermore, both materials are susceptible to fatigue cracking due to cyclic loading from waves, tides, and mechanical operations. These factors reduce the structural life expectancy and increase safety risks over time [9,10]. Such limitations have driven a technological shift in material development and selection, with growing interest in advanced composites that offer better long-term performance, lower weight, and resistance to marine degradation.

### 1.3. Alternative Solutions: Fiber-Reinforced Composites (FRCs)

FRCs present a promising alternative to conventional materials in marine applications. These materials are engineered by embedding high-performance fibers such as glass, carbon, basalt, or aramid into a polymeric matrix, typically thermoset or thermoplastic resins [1,4]. This combination produces a material system with customizable properties that can be tailored for specific performance requirements. FRCs have demonstrated superior performance in corrosion resistance, weight reduction, and mechanical adaptability when compared to metals [5,11]. The use of advanced fibers enables these composites to withstand high stresses while maintaining flexibility and damage tolerance, which is particularly advantageous in wave-impacted marine structures.

**Figure 1:**
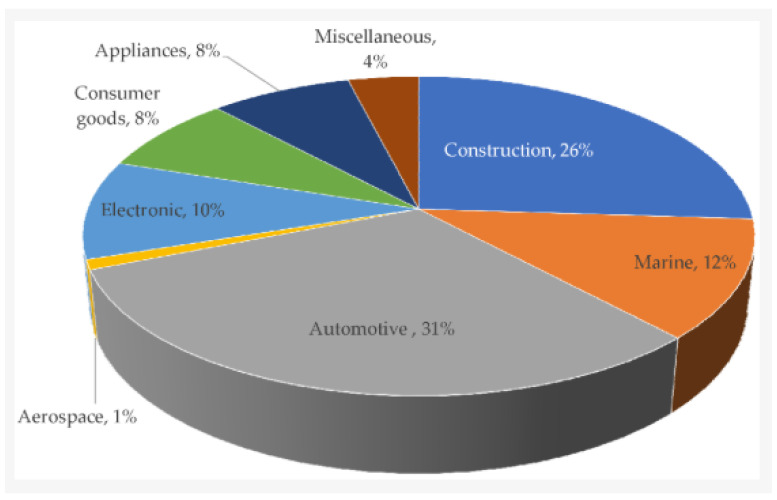
Market share of Fiber-Reinforced Polymer (FRP) by application. Reproduced from [14], MDPI, 2023.

Additionally, FRCs allow for integrating multiple structural functions, such as vibration damping and thermal insulation, into a single component. The ability to tailor composite properties also enables optimization in hulls, pressure vessels, and deck panels where both structural efficiency and durability are paramount [12,13]. As marine systems become more advanced and complex, the adaptability of FRCs provides designers with the freedom to innovate while achieving compliance with stringent performance and safety standards. Currently, the marine sector accounts for approximately 12% of the global market share in fiber-reinforced composite manufacturing, reflecting its increasing adoption in high-performance structural applications (Figure 1) [14].

### 1.4. Key Benefits of FRCs: Lightweight, High Strength, and Corrosion Resistance

The primary appeal of FRCs in marine deck structures lies in their unmatched balance of mechanical and environmental performance characteristics. First and foremost, FRCs are significantly lighter than traditional metals (e.g., steel, aluminum) and exhibit superior corrosion resistance, making them ideal for marine environments. Unlike metals, FRCs are not susceptible to electrochemical degradation, eliminating the need for costly coatings and extensive maintenance procedures in marine environments [4,6]. Moreover, replacement of steel with composites for the manufacture of marine structures will reduce structural weight by 20–40%, directly enhancing vessel fuel efficiency and increasing cargo capacity [1,15]. This weight reduction also simplifies installation and handling, particularly in offshore platforms and large-scale marine modules. Carbon fiber-reinforced polymers (CFRPs), in particular, offer tensile strengths that exceed 3500 MPa, enabling them to rival or surpass the strength of steel without incurring a weight penalty [8,16]. Furthermore, FRCs provide superior fatigue performance under repetitive wave loading conditions, maintaining structural integrity over long periods [9,17]. Finally, their compatibility with advanced manufacturing techniques such as VARTM and automated fiber placement (AFP) enables the creation of complex shapes and integrated designs, further enhancing structural efficiency [18,19].

### 1.5. Importance of Underwater Applications for Marine Decks

Underwater applications impose even more stringent requirements on marine materials, involving high hydrostatic pressure, extreme cold, biofouling, and chemical attack from saltwater. FRCs have emerged as viable candidates for these hostile environments due to their exceptional performance characteristics [20,21]. In offshore oil and gas platforms, FRCs are now used for helidecks, walkways, and risers where their corrosion resistance and light weight reduce maintenance costs and improve structural reliability [2,22]. Submarines and deep-sea installations increasingly rely on carbon fiber composites for pressure hulls, where their high strength and low density contribute to both safety and buoyancy at significant depths [13,23]. In the renewable energy sector, floating platforms for wind turbines have adopted glass fiber-reinforced polymers (GFRPs) for their resilience to constant wave action and saltwater immersion [24,25,26]. Thermoplastic-based FRCs such as those using polyether ether ketone (PEEK) are especially useful for underwater pipelines and buoyancy control modules due to their long-term chemical stability and recyclability [11,27]. These diverse applications illustrate the broad potential of FRCs to revolutionize structural design in underwater marine environments by offering enhanced durability and reduced lifecycle costs.

### 1.6. Significance of the Review

This review aims to bridge existing knowledge gaps by offering a comprehensive assessment of FRCs in marine deck applications, with particular emphasis on underwater conditions. Although previous studies have addressed specific concerns such as corrosion protection, flame retardancy, or fatigue resistance, an integrated analysis encompassing material types, processing methods, and performance under combined marine stressors is notably lacking [6,9,28]. Key contributions of this review include a detailed comparison between thermoset and thermoplastic matrices and the selection of reinforcement fibers such as carbon, glass, basalt, and aramid for marine durability [11,29]. The review also highlights recent innovations in composite manufacturing techniques such as VARTM, AFP, and additive manufacturing (AM), which are enabling the scalable production of large and complex marine components [19,30,31,32]. Furthermore, degradation mechanisms such as seawater-induced aging, moisture ingress, and microbial biofouling are discussed to understand long-term performance challenges [20,21,33]. Finally, the review explores future directions, including the development of self-healing materials, bio-based composite systems, and intelligent coatings that can respond to environmental changes [34,35,36]. By synthesizing these critical areas, the review provides a strategic roadmap for advancing marine deck technologies using next-generation FRCs.

In summary, this review provides a unified evaluation FRCs for marine deck applications through the integration of three key aspects: (1) comparative material performance in submerged environments, (2) scalable manufacturing methods, and (3) degradation mechanisms unique to underwater exposure. In contrast to previous reviews that address these factors separately, this work provides the first comprehensive synthesis linking material selection, processing techniques, and durability considerations—presenting new insights and a strategic foundation for advancing FRC use in demanding marine environments.

**Figure 2:**
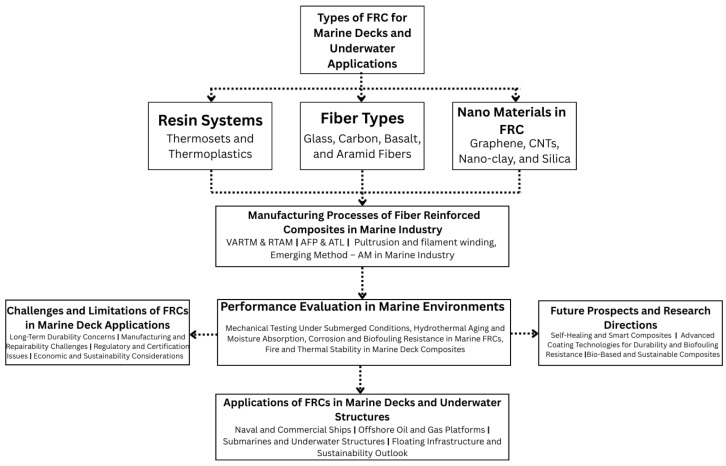
Flowchart of FRC materials, processes, and marine applications.

To easily understand the flow of the paper, Figure 2 illustrates the overall framework of this review, highlighting the interconnections between FRC constituents, manufacturing techniques, performance evaluations, and their marine and underwater applications. A list of abbreviations used in this paper is presented prior to the abstract for clarity.

## 2. Types of FRC for Marine Decks and Underwater Applications

### 2.1. Introduction to Resin Systems in Marine Applications

Resin systems play a pivotal role in marine applications, offering structural integrity, durability, and resistance to harsh environmental conditions [5,37,38]. From traditional thermosetting resins like epoxy and polyester to advanced bio-based and nanocomposite formulations, these materials have revolutionized boatbuilding, offshore structures, and marine coatings [11,39]. Resins serve as the binding matrix, securing reinforcing fibers such as glass, carbon, and aramid in place to ensure structural integrity [4]. The most popular resin transfer systems in marine composites are Resin Transfer Molding (RTM) and VARTM [40,41]. In RTM, resin is injected under pressure into a closed mold containing dry fibers, whereas VARTM uses vacuum pressure to draw the resin into the fiber preform, offering a cost-effective alternative with improved fiber wetting and reduced void content. RTM offers precise resin control, faster cycle times, and high-quality surface finishes, while VARTM provides lower tooling costs, improved fiber wetting, and the ability to produce large, complex structures with minimal voids [12].

**Figure 3:**
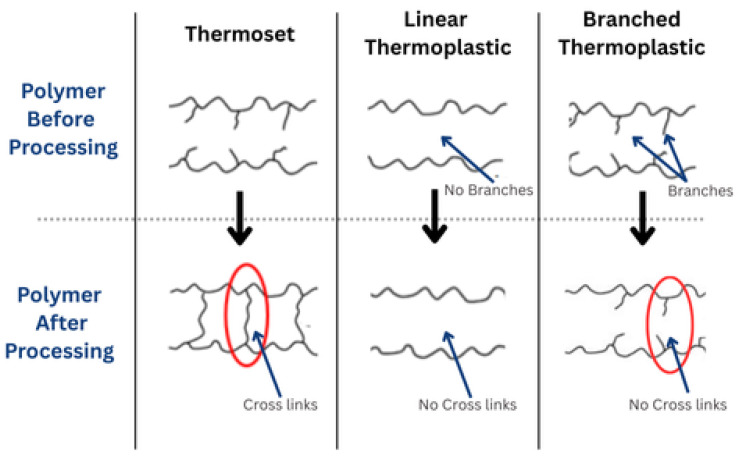
Cross-linked strategy for thermosets and linear or branched strategy for thermoplastics.

Selecting the right resin system is vital for marine environments, where materials face harsh conditions like saltwater corrosion, UV degradation, hydrothermal aging, and mechanical loads. Traditional petroleum-based resins, reinforced with synthetic fibers, are industry standards but pose environmental challenges [42]. The resin matrices used are mainly categorized as thermosetting resin and thermoplastic resin [12]. Their end-of-life (EOL) disposal often involves landfilling or incineration, which conflicts with sustainability goals. This has spurred the development of eco-friendly alternatives, such as bio-based resins and recyclable thermoplastics, which meet structural and functional requirements while reducing environmental impact [42]. Figure 3 illustrates the cross-linked molecular strategy for thermosetting resins and the linear or branched strategy for thermoplastic resins, highlighting their fundamental structural differences and implications for marine applications [43].

#### 2.1.1. Thermoset Resins in Marine Applications

Thermosetting polymers are highly cross-linked macromolecular materials that undergo an irreversible curing process, making them infusible and insoluble [44,45]. Thermosets maintain structural integrity under heat but decompose at elevated temperatures due to bond breakdown. Diglycidyl Ether of Bisphenol A (DGEBA) epoxy, for example, has a thermal decomposition temperature (T_d_) of ~343 °C, often resulting in Carbonization [44,45,46]. The T_d_ varies with hardener type and content, which affects cross-link density and stability. Other thermosets include phenol-formaldehyde, urea-formaldehyde, and unsaturated polyester resins. Epoxies like Bisphenol F, Novolac, Epoxy Phenol Novolac (EPN), Epoxy Cresol Novolac (ECN), and Tetraglycidyl Diaminodiphenyl Methane (TGDDM) offer strong properties. Still, their marine durability remains underexplored, requiring further long-term evaluation in marine environments [47,48]. Thermoset resins dominate the marine industry due to their high rigidity, chemical resistance, corrosion resistance, thermal stability, low water absorption, excellent adhesion, superior mechanical strength, and cost-effectiveness compared to traditional materials such as steel and aluminum [42]. Table 1 presents the key properties of commonly used marine-grade thermoset resins.

Common thermoset resins include vinyl ester, polyester, and epoxy, each offering unique advantages. For example, epoxy provides superior adhesion and chemical resistance, vinyl ester excels in corrosion resistance, and polyester is cost-effective [49,51,60,61]. Their strong bonding with fibers mainly through covalent bonds and secondary interactions, along with their resistance to harsh marine conditions, makes thermosets a preferred choice for marine composites [5,39,50,57,60].

#### 2.1.2. Thermoplastic Resins in Marine Applications

Thermoplastics are linear or branched macromolecular materials that soften and flow when heated, then solidify upon cooling [12,62,63]. This reversible process allows reshaping and recycling. They lack cross-linking, making them flexible, but they can degrade at high temperatures due to polymer chain breakdown [12,62,63,64]. Thermoplastics are gaining attention for their recyclability, reparability, and environmental benefits, such as reduced waste generation, lower lifecycle emissions, and potential for reuse in circular manufacturing systems [65,66].

Thermoplastics for marine applications can be categorized into four main groups: (1) commodity thermoplastics such as Polyethylene (PE) and Polypropylene (PP); (2) engineering thermoplastics including Polyamides (PA) and Polyether Ether Ketone (PEEK); (3) bio-based thermoplastics like Polylactic acid (PLA) and Polyhydroxyalkanoates (PHA) [5,67,68,69]; and (4) high-performance polymers such as PEEK, Polyether Ketone Ketone (PEKK), and Elium™ (developed by Arkema) [42,70]. These materials are particularly valuable for demanding marine environments due to their combination of thermal stability, mechanical strength, and recyclability [11]. The T_d_ of thermoplastics varies with polymer type; for instance, PP decomposes at approximately 300–400 °C, while high-performance thermoplastics such as PEEK exhibit T_d_ values exceeding 575 °C [71,72,73]. These materials can undergo reprocessing and recycling, making them a more sustainable alternative to thermosets, as highlighted in various studies [5,74]. Table 2 presents the key properties of commonly used marine-grade thermoplastic resins.

#### 2.1.3. Comparative Analysis: Thermoset vs. Thermoplastic

Thermoset and thermoplastic composites each offer unique advantages and disadvantages for marine deck and underwater applications. Despite the environmental benefits and recyclability of thermoplastics, their implementation in marine composite applications faces significant challenges [11]. The inherently high viscosity and elevated processing temperatures of thermoplastics demand specialized manufacturing equipment, which increases complexity and production costs. Additionally, achieving strong and durable interfacial bonding between thermoplastic matrices and reinforcing fibers remains a major technical hurdle [11,29,42]. This challenge can negatively impact mechanical performance and long-term durability under harsh marine conditions, such as saltwater exposure and cyclic loading. Consequently, these factors contribute to complex long-term cost-performance trade-offs, underscoring the need for further research to optimize thermoplastic composites for sustainable and effective marine use [11,42,79].

Table 3 provides a comparative analysis of thermoset and thermoplastic resins in terms of manufacturing complexity, recyclability, interfacial bonding, mechanical performance, cost, environmental impact, and shelf life.

### 2.2. Fiber Types

FRCs have significantly transformed marine engineering by offering lightweight, strong, and corrosion-resistant alternatives to traditional materials like steel and aluminum [5,12,75,80]. Marine decks and underwater structures face extreme conditions, including saltwater exposure, mechanical stress, biofouling, and temperature fluctuations. Conventional materials often suffer from corrosion, fatigue, and high maintenance costs, making FRCs a superior choice for long-term durability and performance [4,12,83,84].

**Figure 4:**
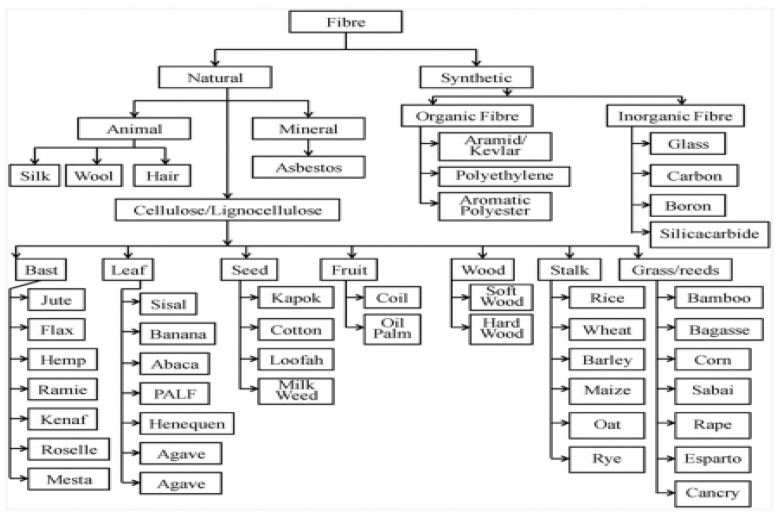
Classification of fibers. Reproduced from [91], Wiley, 2024.

FRCs consist of high-performance fibers embedded in a polymer matrix, enhancing strength while reducing weight. The selection of an appropriate fiber type is critical for ensuring optimal mechanical properties, environmental resistance, and cost-effectiveness in marine applications [85,86,87]. Key factors influencing fiber selection include tensile strength, moisture absorption, thermal stability, impact resistance, and long-term durability [84,88,89,90]. Figure 4 shows the general classification of fibers used in FRCs, providing a framework for selecting the appropriate fiber for marine environments.

Among the most commonly used fibers in marine applications are glass, carbon, basalt, and aramid fibers.

#### 2.2.1. Glass Fiber-Reinforced Polymers in Marine Applications

Glass fiber is a widely used reinforcement material in marine applications due to its high strength-to-weight ratio, corrosion resistance, and cost-effectiveness. It is primarily composed of silica (SiO_2_), with varying proportions of alumina (Al_2_O_3_), calcium oxide (CaO), magnesium oxide (MgO), and boron oxide (B_2_O_3_) [92,93,94,95,96,97]. The manufacturing process involves melting raw materials at 1300–1500 °C, followed by extrusion through fine orifices to form continuous filaments [98,99]. These filaments are coated with sizing agents to enhance adhesion with polymer matrices and improve handling during processing. Glass fibers are available in various forms, including rovings, chopped strands, and woven fabrics, depending on the intended application [96,98,100].

The two most common types of glass fibers used in marine applications are E-glass and S-glass. E-glass offers good mechanical properties, electrical insulation, and resistance to moisture and corrosion, making it suitable for general marine structures [1,96,98]. S-glass, with its higher tensile strength and stiffness, is used in high-performance applications requiring enhanced mechanical properties. These fibers are typically embedded in thermoset resin systems such as epoxy, vinyl ester, or polyester to improve their durability and resistance to hydrothermal degradation. Epoxy resins offer strong adhesion and superior chemical resistance, vinyl ester resins provide excellent water resistance and impact strength, and polyester resins serve as a cost-effective alternative with moderate durability and mechanical performance [37,61,96,100,101,102]. In addition to thermosets, thermoplastic resins are also increasingly being explored in marine composites for their recyclability, faster processing, and toughness. Examples include PP, PA, and high-performance options like PEEK, which offer excellent moisture resistance and mechanical stability in demanding marine environments [26,74,103,104]. Though less common than thermosets in current marine applications, thermoplastics are gaining attention due to their potential for long-term sustainability and repairability [74,103,104].

Glass fiber composites are used extensively in marine applications, including small boats, ship hulls, decks, and bulkheads, where their corrosion resistance and lightweight properties provide structural advantages [1,61,74,96]. They are also applied in offshore platforms, underwater pipelines, and buoy systems, offering durability in harsh marine environments [2,54,102,105].

#### 2.2.2. Carbon Fiber-Reinforced Polymers in Marine Applications

Carbon fiber is a high-performance reinforcement material extensively used in marine applications due to its exceptional strength-to-weight ratio, stiffness, and fatigue resistance [1,8,16]. Its crystalline structure, composed of carbon atoms, provides high tensile strength (3500–7000 MPa) at a low density (1.6–1.8 g/cm^3^), making it ideal for lightweight yet durable marine structures [16,106,107].

Carbon fibers are classified into high-strength (HS), intermediate-modulus (IM), and high-modulus (HM) types, each offering specific benefits [1,97,108,109,110]. HS fibers provide excellent tensile strength for general marine structures, while IM fibers balance strength and stiffness for load-bearing applications. HM fibers, with superior rigidity, are used in advanced structural components requiring minimal deformation under stress [97,108,110,111]. Unlike glass fibers, carbon fibers exhibit lower moisture absorption, reducing hydrothermal degradation risks in prolonged seawater exposure [108,112]. These fibers are most commonly used with thermoset resins such as epoxy, vinyl ester, and polyester [16,107,112,113]. Epoxy resins are the most widely used due to their excellent adhesion, chemical resistance, and low moisture absorption [107,111,112,113]. Vinyl ester resins provide good water resistance and impact strength at a lower cost, while polyester resins are more affordable but less durable, making them suitable for non-structural parts [112,113,114]. In addition, thermoplastic resins such as PEEK and PA are gaining traction for carbon fiber composites [103,115,116,117,118,119]. These resins offer high toughness, rapid processing, and recyclability, along with excellent resistance to moisture and chemicals, making them promising for next-generation marine structures, especially where repairability and environmental sustainability are priorities [103,115,117,118,120].

Marine applications of CFRPS include ship hulls, naval vessels, and deep-sea submersibles, where lightweight yet strong materials enhance performance and fuel efficiency [1,107,108,111]. Additionally, carbon fiber is used in propeller blades, hydrofoils, end fittings, and offshore wind farm structures, improving hydrodynamic efficiency and durability [8,108,112].

#### 2.2.3. Basalt Fiber-Reinforced Polymers in Marine Applications

Basalt fiber is a high-performance reinforcement material derived from natural basalt (volcanic) rock, offering superior mechanical properties, chemical resistance, and thermal stability for marine applications. The production process involves melting basalt rock at 1400–1500 °C, followed by extrusion through fine nozzles to create continuous filaments [121,122,123]. These filaments are then processed into rovings, mats, or woven fabrics for various engineering applications [121,122,123]. Unlike glass fibers, basalt fibers are produced without the addition of harmful additives such as boron or other toxic chemicals, making them more environmentally friendly and safer to manufacture [123,124,125,126,127]. Their high thermal resistance, up to 600 °C, and monofilament strength ensure excellent performance in extreme environments such as engine components [123,125,128].

Basalt fibers are classified by processing method and application. Continuous basalt fibers are used in advanced composites, while chopped fibers reinforce concrete [121,123,128]. Woven basalt fabrics add structural integrity in load-bearing uses [121,123]. In composite manufacturing, basalt fibers are typically paired with thermoset resin systems such as epoxy, vinyl ester, and polyester, similar to carbon fiber systems. Among these, epoxy resins are widely preferred due to their strong adhesion, chemical resistance, and superior mechanical properties. Vinyl ester resins offer good water resistance and toughness, while polyester resins present a cost-effective solution for less demanding applications [122,123,124,125,127]. Thermoplastic resins such as PA, PP, and PEEK are being increasingly explored in basalt fiber composites [129,130,131]. These thermoplastics provide advantages like recyclability, impact resistance, and faster processing, making them suitable for automotive, construction, and marine industries where sustainability and performance are critical [129,131].

Marine applications of basalt fiber composites include naval vessels, offshore platforms, and underwater pipelines. Their corrosion resistance makes them ideal for ship hulls, safety decks, and reinforcements in offshore oil rigs [121,122,123,125,132]. Additionally, basalt fibers strengthen concrete structures in floating platforms and mooring systems, ensuring long-term durability in harsh marine conditions [121,123,125,127,132].

#### 2.2.4. Aramid Fiber-Reinforced Polymers in Marine Applications

Aramid fibers are synthetic polymers produced through the polymerization of aromatic diamines and acid chlorides [133,134,135]. Their highly crystalline molecular structure contributes to favorable mechanical properties, including high tensile strength (up to 3600 MPa), low density (1.44 g/cm^3^), and good thermal stability [133,134,135,136,137]. These characteristics make aramid fibers suitable for use in marine environments, particularly where components are subject to dynamic loading, fatigue, and abrasion [134,136,137].

Aramid fibers are broadly classified into para-aramids (e.g., Kevlar, Twaron) and meta-aramids (e.g., Nomex), each with distinct structural and performance attributes [135,137,138,139]. Para-aramids are commonly used in structural reinforcements due to their higher tensile strength, while meta-aramids are valued for their flame and thermal resistance in protective applications [137,139]. In composite applications, aramid fibers are typically embedded in thermoset resin systems such as epoxy, vinyl ester, and polyester [134,137,138,139]. Epoxy and polyester offer good mechanical properties and adhesion, vinyl ester provides moisture resistance, and sizing agents are often applied to improve fiber-matrix interaction [134,137,139,140]. Emerging applications also employ thermoplastic resins such as PEEK and PA with aramid fibers [103,118,120,141,142]. These materials offer better toughness, faster processing times, and recyclability, making them increasingly attractive for lightweight, high-performance composites in aerospace, defense, and marine applications [103,120,142].

In marine applications, aramid FRCs are used in structural and protective components, including boat hulls, flywheel cables, safety nets, and coatings for impact- and fatigue-prone areas [134,137]. The specific performance requirements, material costs, and environmental conditions determine their use [134,136,137,140].

Table 4, Table 5 and Table 6 provide a comprehensive overview of fiber materials used in composite manufacturing. Table 4 summarizes the main types of fibers, outlining their general characteristics and applications. Table 5 lists commonly used fibers in composite structures, particularly relevant to marine engineering. Table 6 presents a critical comparison of these fiber types for marine applications, evaluating their performance, durability, and suitability under varying operational conditions.

### 2.3. Nano Materials in FRC

Nanomaterials have become pivotal in advancing marine composites, offering substantial improvements in mechanical properties, thermal stability, and functional performance [143,144]. Four key nanomaterials dominate current applications: graphene and carbon nanotubes (CNTs), which enhance fracture toughness and introduce electrical conductivity [144,145,146,147,148,149]; nano-clays, which improve Fire Resistance and barrier properties via winding mechanisms [150,151,152]; and nano-silica, which increases resin stiffness and reduces water uptake by up to 25% through strong matrix interactions [145,151,152,153,154].

These nanomaterials significantly enhance composite durability when incorporated into thermoset resins such as epoxy and vinyl ester [147,150,151,155,156]. For example, nano-silica modifications can raise the glass transition temperature (T_g_) by 10–15 °C while reducing moisture absorption [157,158].

Nanomaterials provide exceptional reinforcement at the fiber–matrix interface [148,150,151,157,159]. Surface-functionalized fibers (e.g., CNT-coated glass or carbon fibers) exhibit up to a 40% increase in interfacial shear strength due to improved chemical bonding and mechanical interlocking [147,148,152,155,156]. This enhancement directly enhances fatigue resistance and impact performance—critical properties for marine structures subjected to cyclic loads and dynamic stresses [147,148,152].

Beyond structural enhancements, nanomaterials also enable smart functionalities. CNT and graphene networks facilitate real time strain sensing, essential for early damage detection in inaccessible or submerged marine environments [148,152,156,160,161]. Antimicrobial nanoparticles (e.g., nano-ZnO) reduce biofouling [162], while nano-clays and aluminum hydroxide (Al(OH)_3_) enhance fire resistance by delaying ignition and reducing heat release rates [160,163].

Table 7 lists nanomaterials’ key properties, loadings, challenges, and marine uses like coatings, hulls, and protective structures.

However, there are still some challenges in processing, particularly related to the resin thickness. Adding nanoparticles can make the resin thicker, which makes it harder to use in processes like VARTM [164].

### 2.4. Manufacturing Processes of Fiber-Reinforced Composites in the Marine Industry

The marine industry has witnessed a transformative shift toward the use of composite materials due to their superior strength-to-weight ratio, corrosion resistance, and design adaptability [1]. The effectiveness of marine composites is closely tied to the manufacturing process, which directly influences fiber alignment, void content, and the overall structural integrity of the final component [30,165]. Several manufacturing techniques have emerged or evolved to meet the performance demands and sustainability goals of modern marine applications. Among these, common manufacturing processes like VARTM, RTM, AFP, Automated Tape Laying (ATL), pultrusion, filament winding, and emerging methods of AM represent key methodologies tailored for different component geometries and material types [30,165,166].

#### 2.4.1. Common Manufacturing Processes in the Marine Industry

i. VARTM and RTM

VARTM and RTM remain dominant in the production of large-scale marine structures such as hulls and decks. VARTM operates by using vacuum pressure to draw resin into dry fiber preforms placed within a single-sided mold [18,167,168,169]. Its cost-effectiveness and scalability make it suitable for manufacturing large, low-cost components [31,170,171]. However, precise control of resin viscosity, flow rate, and fiber compaction is critical to minimizing voids and ensuring consistent quality [18,31,167,169,170]. In contrast, RTM uses a closed mold with injected resin under pressure, enabling better fiber wet-out, reduced porosity, and enhanced surface finish [18,168,170,172]. These methods are highly compatible with thermosetting resins, especially epoxy, vinyl esters, and polyester systems, which exhibit strong mechanical properties and chemical resistance [18,167,168,173,174]. Also, RTM and VARTM have shown compatibility with nano-enhanced resins such as those embedded with CNT or nano-silica, although challenges persist regarding nanoparticle dispersion and resin viscosity management [143,172].

ii. AFP and ATL

Automated manufacturing processes such as AFP and ATL have brought significant innovation to the production of high-performance marine composites [15,175]. AFP is especially beneficial for laying narrow prepreg tows along complex contours with high precision, allowing for controlled fiber orientation and improved load-bearing efficiency [19,176,177]. ATL, while similar, uses wider composite tapes and is better suited to planar or slightly curved surfaces and faster material deposition than AFP [15,19,178]. These processes, initially optimized for thermoset matrices, have recently been adapted to accommodate thermoplastics, enabled by advanced heating systems such as infrared or laser-assisted consolidation [15,19,176,178]. The application of thermoplastics, particularly high-performance types like PEEK and PEKK, in these automated systems offers the dual advantage of recyclability and rapid processing cycles [177,178]. These properties are highly desirable in applications where lightweight structures, mechanical performance, and reusability are critical, such as in naval defense and high-speed vessels [175,178,179].

iii. Pultrusion and filament winding

Pultrusion and filament winding are continuous fabrication processes widely used for producing marine components with constant cross-sections [166,180,181]. In pultrusion, continuous fibers are pulled through a resin bath and shaped within a heated die, forming strong, uniform profiles such as rods, beams, and pipes [166,180,181,182,183]. Filament winding, by contrast, involves winding resin-impregnated fibers around a rotating mandrel to form hollow, pressure-resistant structures such as tanks or masts [166,181,184,185]. Both processes ensure high fiber volume fractions and are increasingly being adapted for thermoplastic resins, broadening their application in recyclable composite structures [180,181,184,186,187]. Despite technical challenges, nanomaterial integration into these methods enhances mechanical strength, fatigue resistance, and anti-corrosion performance—attributes essential for long-term marine durability [181,185].

#### 2.4.2. Emerging Method—AM in the Marine Industry

AM represents a more recent development in composite fabrication, offering a layer-by-layer approach to creating complex geometries with minimal waste [32,188,189]. Techniques such as fused deposition modeling (FDM) and continuous fiber-reinforced 3D printing are under investigation for marine applications, particularly for prototyping, low-volume customized components, and on-site repairs [32,188,189,190]. However, technical limitations such as reduced mechanical strength due to anisotropic properties, fiber discontinuity, and insufficient resin infiltration restrict their use in critical load-bearing structures [32,188,191,192]. Thermoplastic resins like PA, PEEK, and PP are commonly used due to their processability [188,189,193,194], recyclability, and compatibility with AM techniques [189,190,194]. Research efforts are ongoing to improve these technologies through material innovation and hybrid manufacturing approaches that combine additive and conventional methods [32,91,190,195].

Recent research highlights the promising potential of AM to revolutionize fiber-reinforced composites for marine decks. Unlike conventional methods like hand layup or VARTM, AM enables on-demand production of complex, lightweight geometries with minimal waste, ideal for customized components and onboard repairs in the marine industry [188,196]. However, challenges like anisotropic strength and interlayer adhesion persist, driving hybrid approaches or overprinting onto preforms [197]. Recent investigations also explore optimizing fiber orientation paths through topology-guided algorithms to tailor stiffness and strength distributions within printed parts [179,189,192]. Additionally, Multi Jet Fusion (MJF) and in-situ monitoring technologies, including thermal imaging and embedded sensors, are being developed to ensure consistent quality during AM processes and detect defects early [142,196,198]. Emerging advances in AI-driven process optimization and sustainable materials (e.g., recycled fibers or bio-based resins) could accelerate AM’s adoption, positioning it as a transformative solution for next-generation maritime structures and contributing to more efficient, sustainable shipbuilding practices [189,196].

Table 8 outlines composite manufacturing methods, detailing processes, advantages, limitations, and marine industry applications.

## 3. Performance Evaluation in Marine Environments

### 3.1. Mechanical Testing Under Submerged Conditions

Marine-grade FRCs are critical materials for offshore and underwater applications, where they must retain structural integrity under continuous seawater immersion, thermal cycling, and mechanical loading [3,20,200,201]. The mechanical behavior of these composites under submerged conditions has been extensively studied through standardized testing methods and advanced simulation techniques [20,202,203]. Comparative studies of thermoset composites such as epoxy and polyester against thermoplastic composites like PEEK, PEKK, and Elium™ demonstrate distinct differences in degradation mechanisms and long-term durability [29,204].

**Figure 5:**
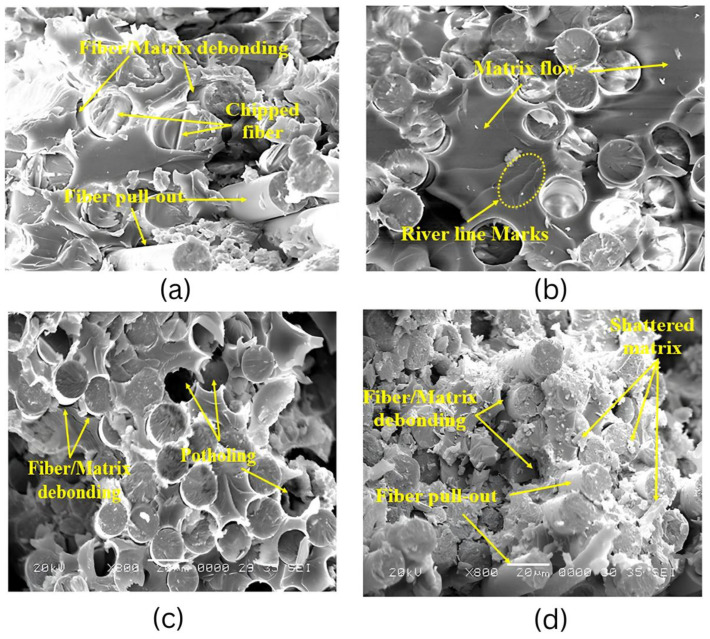
Fracture surfaces of the E-glass/epoxy composite conditioned for 5 years at (**a**) 23 °C, (**b**) 65 °C, Fracture surfaces of the E-glass/epoxy composite conditioned for 11 years at (**c**) 23 °C, (**d**) 65 °C, Reproduced from [213], MDPI, 2021.

**Figure 6:**
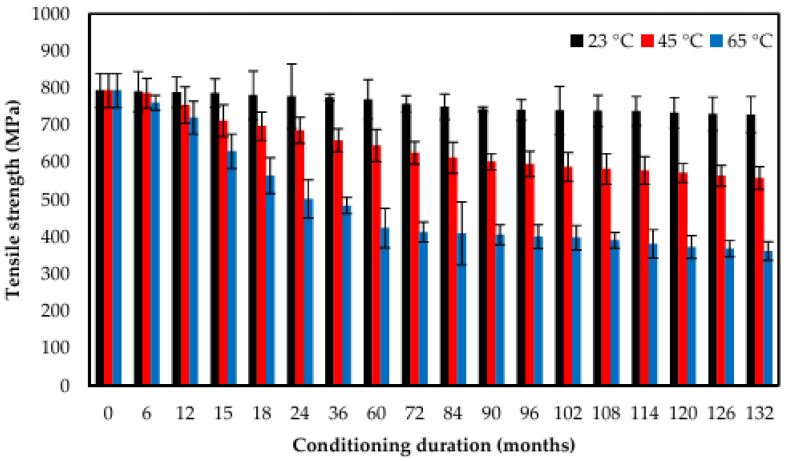
Tensile strength versus exposure time at different temperatures for E-glass/epoxy composite, Reproduced from [213], MDPI, 2021.

Tensile testing is a fundamental method for evaluating the strength and stiffness of composites exposed to marine environments. Standards such as ASTM D3039 for fiber-reinforced composites and ASTM D638 [205] for unreinforced plastics have been utilized in various studies; however, ASTM D3039 [206] is predominantly used for assessing the mechanical performance of structural composites under submerged or seawater-aged conditions [200,207,208,209]. These tests are critical in understanding how seawater immersion influences tensile behavior, primarily through mechanisms such as matrix plasticization, hydrolysis, and fiber–matrix debonding [60,210,211]. As shown in Figure 5 and Figure 6, the fracture surface morphology and tensile strength over time at different temperatures illustrate the long-term degradation behavior of E-glass/epoxy composites under marine conditioning. Thermoset composites, particularly those based on epoxy/glass and epoxy/carbon systems, typically retain between 70% and 90% of their initial dry tensile strength after 6 to 12 months of continuous immersion in seawater environments. However, this retention can vary depending on environmental conditions, material composition, temperature, salinity, and immersion duration [204,212,213,214,215].

Vinyl ester-based composites exhibit slightly improved retention due to their lower water uptake and enhanced chemical resistance [216,217]. Thermoplastic composites, particularly those reinforced with carbon fibers and based on PEEK, demonstrate the highest retention of mechanical properties, often exceeding 90% after prolonged exposure to seawater and submerged environments [11]. This exceptional durability is attributed to the hydrophobic nature of the PEEK matrix and its superior resistance to hydrothermal degradation, which effectively limits moisture uptake and preserves fiber–matrix interfacial strength [27,218].

**Figure 7:**
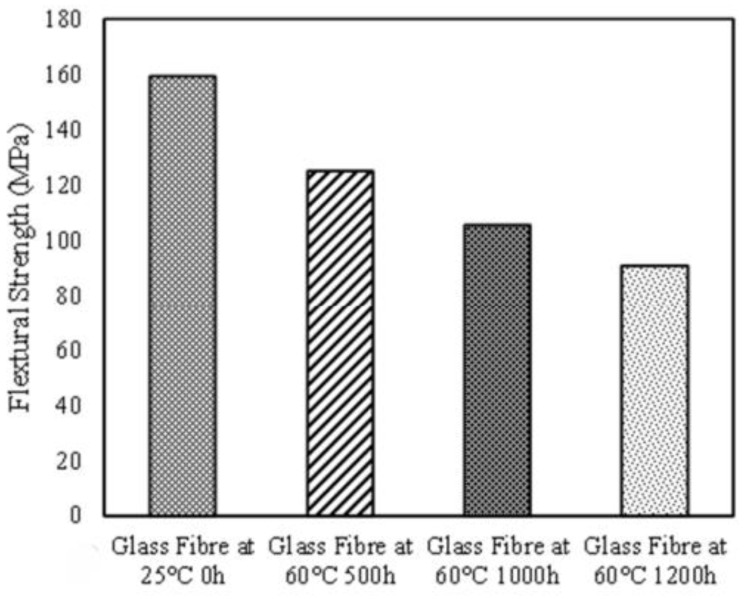
Flexural strengths of GFRE samples change with temperature and duration. Adapted from [88], MDPI, 2022.

The most common test types used to evaluate the bending behavior of fiber-reinforced polymer composites are Flexural Testing following ASTM D7264 [219], ASTM D790 [220], and ISO 14125 [221]. These tests assess a material’s resistance to bending deformation and its ability to maintain stiffness under mechanical load [205,209,220,221,222,223]. In marine and hygrothermal environments, absorbed moisture plasticizes the matrix, reduces the interlaminar shear strength, and increases the likelihood of delamination, especially under cyclic or sustained flexural loading [204,224,225,226]. Epoxy/carbon fiber laminates are known to retain approximately 75–85% of their dry-state flexural modulus after long-term immersion in seawater at elevated temperatures [227,228]. This retention is attributed to the low moisture absorption of carbon fibers and the relatively stable performance of epoxy resins [6,229]. In contrast, composites reinforced with glass or basalt fibers tend to exhibit more significant reductions in flexural properties due to higher fiber–matrix interfacial degradation in moist environments [214,230]. The flexural response of a composite is influenced by multiple factors, including the type of matrix resin, the nature of the reinforcing fibers, the fiber volume fraction, and the layup architecture (e.g., cross-ply vs. unidirectional). Additionally, environmental conditioning parameters such as temperature, exposure duration, and salinity of the immersion medium also play critical roles in governing degradation behavior [204,212,213,224,231]. This trend is illustrated in Figure 7, which shows the reduction in flexural strength of glass fiber-reinforced epoxy (GFRE) samples as a function of both exposure temperature and duration [88].

Impact testing is essential for evaluating how marine composites withstand sudden dynamic loads like wave impacts or collisions. Standard methods such as ASTM D256 [232], Zwick/Roell Charpy impact testing (Izod/Charpy) [233], and the CEAST 9350 (Fractovis Plus) impact test machine [234] (drop-weight) reveal that seawater exposure degrades impact resistance through three key mechanisms: microcracking, fiber–matrix debonding, and resin plasticization [20,235]. Studies show GFRP loses 20–40% impact strength after immersion for 6–12 months, while CFRP fares better with only a 10–20% reduction due to their hydrophobic nature [214,227]. Aramid fiber composites demonstrate an exceptional performance, retaining their dry-state impact strength because of the fibers’ inherent toughness and low moisture absorption [136,236]. Recent advances using nanofillers like CNTs show promise, improving wet-state impact resistance by 25–30% through enhanced crack deflection and interfacial bonding [203,237,238]. However, real-world effectiveness depends on proper nanoparticle dispersion, which remains a manufacturing challenge [237,239]. Environmental factors significantly influence degradation rates. Elevated seawater temperatures (65 °C vs. 23 °C) accelerate strength loss by nearly twice the amount, while cyclic wet-dry exposure causes more severe damage than continuous immersion [208,213,240]. These findings highlight the need for material selection based on specific service conditions, with hybrid composites and nano-modified resins offering potential solutions for critical marine applications requiring long-term durability [237,241,242].

Fatigue resistance represents a critical performance parameter for marine composites subjected to cyclic loading in seawater environments. Standardized testing according to ASTM D3479 [243] and ISO 13003 [244] reveals substantial differences in fatigue behavior between material systems [9,245]. Glass fiber-reinforced thermoset composites typically exhibit 30–50% reductions in fatigue life following prolonged seawater exposure, with degradation mechanisms including matrix plasticization, fiber–matrix interface weakening, and accelerated crack propagation [10,246,247,248]. In contrast, carbon fiber composites demonstrate superior performance, particularly when paired with thermoplastic matrices such as PEEK, showing less than 20% fatigue life reduction under comparable conditions [17,239,249,250]. This enhanced durability stems from carbon fibers’ inherent hydrophobicity and the superior moisture resistance of thermoplastic resins [250]. Aramid fiber composites display exceptional fatigue-impact synergy, maintaining 80–85% of their dry-state performance, while emerging basalt fiber systems offer a cost-effective intermediate solution [21,122,136,251]. Recent advances in nanocomposite modification, particularly through the incorporation of 0.3–0.7 wt% CNTs, have shown potential to improve fatigue resistance by up to 250% in epoxy-based systems [252,253,254].

A comparative evaluation of these mechanical tests reveals clear trends in submerged performance. Epoxy/glass composites demonstrate moderate retention across most metrics but suffer significant impact and fatigue degradation [10,209,246]. Vinyl ester/glass systems outperform epoxies in wet tensile and flexural tests [216,255]. Carbon fiber-based composites deliver better stiffness and fatigue performance than their glass counterparts, while thermoplastics consistently provide the best overall mechanical retention across all test types [17,249].

**Figure 8:**
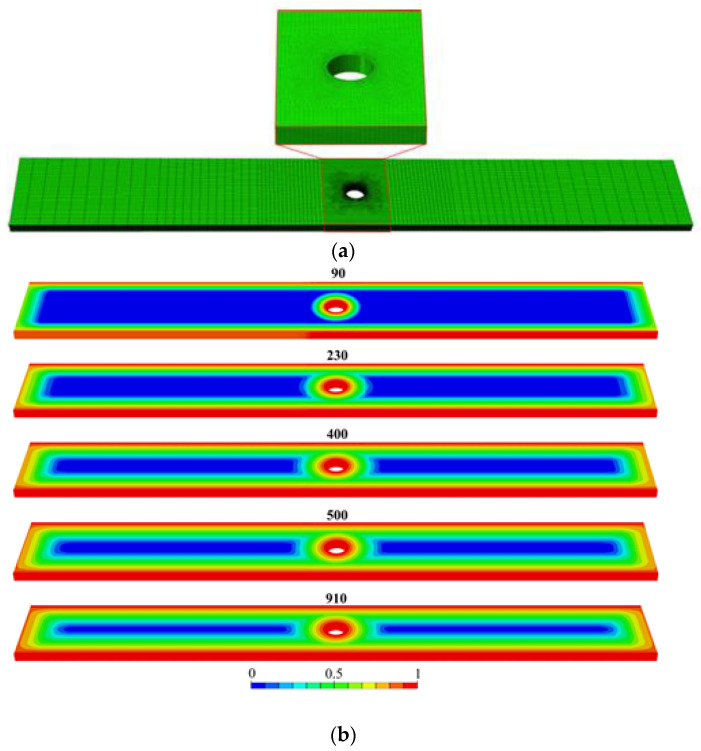
(**a**) Finite element mesh used in the finite element analysis, (**b**) Numerical prediction of the sea-water concentration for several days, Adapted from [246], Elsevier, 2024.

Recent advances in finite element analysis (FEA) enhance the interpretation of fatigue behavior in composites exposed to seawater by simulating moisture diffusion, stress evolution, and microcrack propagation. FEA-based moisture transport models predict stress concentrations from differential swelling in laminates, correlating with hydrothermal aging and stiffness degradation. This supports experimental results, where fatigue life decreased with longer immersion times [223,256]. For instance, GFRP specimens immersed for 230 and 910 days showed fatigue life reductions of 66% and 95%, respectively, at 143 MPa cyclic stress. Despite this, similar S-N curve slopes suggest consistent failure mechanisms such as matrix cracking and delamination. The fatigue strength dropped from 125.9 MPa (dry) to 107.2 MPa (910 days), aligning with FEA predictions of water uptake and internal damage. For example, Figure 8a shows the finite element mesh used in the analysis, while Figure 8b presents the numerical prediction of seawater concentration over several days; both support the simulation of moisture diffusion relevant to fatigue degradation. Thus, integrating FEA with fatigue testing improves understanding of long-term performance and supports marine-grade composite design [246].

**Figure 9:**
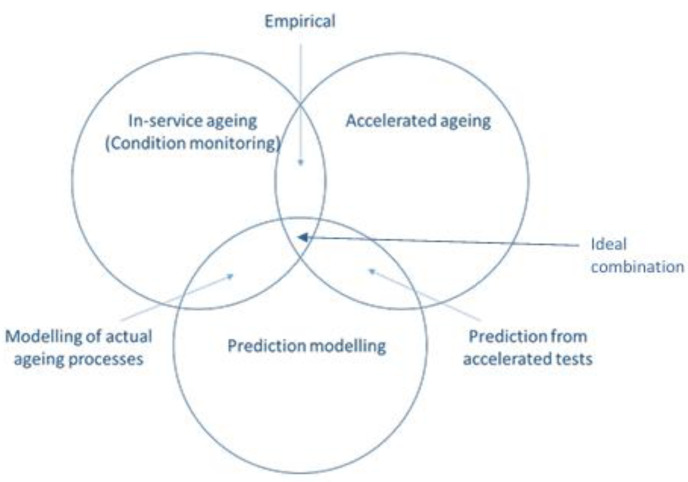
The effect prediction of long-term properties requires a combination of condition monitoring, Reproduced from [256], National Physical Laboratory, 2017.

Best testing practices include accelerated aging protocols (e.g., ASTM D5229 [257]), which combine an elevated temperature and humidity to simulate long-term exposure within a condensed timeframe [225,258]. In-situ submerged test rigs allow for real-time monitoring of mechanical behavior during immersion, providing data that better represents service conditions (Figure 9). Furthermore, post-failure microstructural evaluations using scanning electron microscopy (SEM) and Fourier-transform infrared spectroscopy (FTIR) offer insights into failure mechanisms such as matrix cracking, fiber pull-out, and chemical degradation [220,225,246,256].

### 3.2. Hydrothermal Aging and Moisture Absorption

Moisture uptake in polymer matrix composites under hydrothermal conditions plays a critical role in defining long-term durability, especially in marine environments [246,248,259]. The absorption behavior is broadly governed by diffusion kinetics, typically categorized as either Fickian or non-Fickian [211,224,260]. Fickian diffusion is common in semi-crystalline thermoplastics such as PEEK and PEKK, where moisture uptake follows Fick’s second law of diffusion [248,260,261]. These materials exhibit linear absorption behavior proportional to the square root of time and show very low moisture saturation levels usually below 0.5 wt% due to the restricted movement of water molecules in tightly packed crystalline regions [224,229].

In contrast, thermoset polymers such as epoxy and polyester frequently deviate from Fickian models. Their amorphous nature allows for significant polymer chain relaxation, microcrack formation, and capillary water transport along fiber–matrix interfaces [260,262]. This results in non-Fickian, two-stage absorption behavior characterized by higher saturation levels (typically 4–6 wt%) and more complex, time-dependent kinetics. These degradation phenomena are exacerbated in marine environments due to humidity, elevated temperatures, and cyclic thermal loading [113,263].

The effects of hydrothermal aging on mechanical properties are well-documented in the literature. Interlaminar shear strength (ILSS), a critical parameter for layered composites, is susceptible to prolonged moisture exposure [112,212,226]. Epoxy–carbon fiber systems can lose 20–40% of ILSS after 12 months of immersion in seawater, primarily due to hydrolytic degradation and progressive fiber–matrix debonding [20,216]. In contrast, thermoplastics like PEEK maintain over 90% of their initial ILSS after similar exposure durations, benefiting from their hydrophobic chemical structure and stable semi-crystalline morphology [264,265].

Another significant consequence of hydrothermal exposure is the reduction in T_g_, which can severely limit operational performance [204,225]. For instance, dynamic mechanical analysis (DMA) studies demonstrate that epoxy matrices in fiber-reinforced composites can exhibit a T_g_ reduction of up to 24 °C due to hygrothermal plasticization, shifting from 180 °C (dry) to 156 °C under high humidity conditions (80 °C/95% RH) [266]. PEEK, however, makes it particularly suitable for structural components exposed to thermal cycling, such as marine decks and hulls [267].

To evaluate the long-term performance, several studies have utilized accelerated aging protocols such as those defined in ASTM D5229 combined with finite element simulations [225,258]. Experimental studies on commercial glass fiber-reinforced vinyl ester composites demonstrate significant mechanical degradation under fluid exposure, with flexural modulus reductions of 20–30% after immersion for 6 months in seawater, biodiesel, or oily water at ambient temperature [268]. Finite element analyses using tools like COMSOL Multiphysics v6.1 and ABAQUS 2023 have been employed to model moisture–mechanical coupling and predict service life in marine environments [200,215]. These simulations suggest that an Accurate 3D FEM model predicts seawater degradation in GFRP with a <9% error, validated via Digital Image Correlation (DIC) strain fields and Puck’s failure theory, depending on environmental conditions and structural configurations [200].

Recent developments point toward promising strategies to improve hydrothermal durability. These include the incorporation of self-healing coatings embedded with microencapsulated hydrophobic agents, which aim to restore barrier properties in thermoset matrices [269]. Additionally, machine learning techniques are increasingly being integrated with finite element models to enable more accurate, multi-scale predictions of moisture-induced damage progression and mechanical degradation [259].

### 3.3. Corrosion and Biofouling Resistance in Marine FRCs

FRCs exhibit superior corrosion resistance compared to traditional metals in marine environments; however, their long-term durability remains challenged by electrochemical degradation and biological fouling [6,270]. Three key factors primarily influence these degradation mechanisms: (1) fiber conductivity, where carbon fibers can accelerate galvanic corrosion when coupled with metals due to their high electrical conductivity, whereas glass and basalt fibers are electrically insulating and thus corrosion-resistant, (2) matrix chemistry, where vinyl ester resins offer significantly better hydrolytic stability compared to unsaturated polyesters, and (3) environmental conditions, including salinity, thermal cycling, and ultraviolet (UV) radiation, which collectively exacerbate degradation of fiber-reinforced polymer composites [271,272,273]. Table 9 outlines galvanic corrosion risk, biofouling rate, and effective protection methods for various marine fiber-reinforced composites.

Electrochemical impedance spectroscopy (EIS) reveals that CFRPs lose 60–80% of coating resistance and exhibit a threefold rise in double-layer capacitance after six months of seawater immersion, indicating progressive interfacial delamination and matrix degradation [6].

Biofouling introduces an additional degradation pathway, progressing through three well-defined stages: formation of an organic conditioning film (<1 h), microbial biofilm development (1–7 days), and macrofouling by organisms such as barnacles and mussels (within weeks) [7,33,274,275]. Beyond increasing hydrodynamic drag, biofouling can exacerbate localized corrosion by altering the microenvironment at the FRC surface [275]. Various antifouling coatings offer different performance trade-offs: nano-ZnO/polyurethane systems generate reactive oxygen species and achieve 99% bacterial reduction but suffer from UV-induced degradation; graphene-epoxy coatings reduce barnacle adhesion by 80% but are limited by high cost; and silicone-based foul-release coatings lower cleaning effort by 60% though they exhibit poor abrasion resistance [36,276,277].

### 3.4. Fire and Thermal Stability in Marine Deck Composites

Marine composite structures face distinct fire safety challenges due to their confined underwater environments and exposure to both thermal and hydrothermal stresses [28]. Ensuring fire resistance in applications like submarine compartments, offshore platforms, and vessel components is critical [278,279]. Traditional flame-retardant approaches often rely on mineral fillers such as aluminum trihydroxide (ATH) and magnesium hydroxide (MDH), which suppress fire through endothermic decomposition [278,280]. However, their high loading levels can degrade mechanical properties [279]. Phosphorus-based systems, particularly ammonium polyphosphate (APP)-modified vinyl esters and epoxies, offer enhanced performance in humid conditions by forming robust char layers [280,281]. When combined with nanoclays, these systems also reduce smoke rate by up to 30%, improving safety during evacuation [28,278].

Thermoplastic composites such as PEEK show excellent inherent fire resistance, with peak heat release rates around 125 kW/m^2^ and long ignition delays. Their aromatic molecular structure promotes char formation and minimizes toxic smoke [282,283]. Advances in nanotechnology, like the incorporation of CNTs in epoxy matrices, enhance flame inhibition and maintain structural integrity at high temperatures [284,285].

Sustainable fire-retardant innovations include bio-based intumescent coatings derived from marine biomass, such as chitosan (from crustacean shells), alginate (from brown seaweed) and lignin (a natural polymer from marine and terrestrial plant sources) [286,287,288]. These coatings offer comparable performance to synthetic alternatives by forming thermally stable char layers [281,289,290]. These bio-based coatings provide an eco-friendly solution by reducing environmental impact and enhancing biodegradability, making them especially suitable for marine and construction applications where sustainability is a priority [288,291]. Hybrid ceramic-polymer systems are particularly effective for buoyant and pressure-resistant structures. Looking forward, multifunctional materials that merge flame retardancy with mechanical and hydrothermal resilience are becoming central to design [292,293]. The synergy found in hybrid ceramic-polymer systems combines ceramic robustness with polymer flexibility and corrosion resistance, enabling enhanced flame retardancy alongside structural durability under harsh conditions [292,294]. Research is increasingly focused on smart coatings, optimized nanomaterial dispersions, and real-time monitoring systems, paving the way for safer and more sustainable marine composites [281,293]. Advances in nanomaterial optimization and the integration of real-time damage detection further enhance the multifunctionality and longevity of these composites, ultimately supporting safer, cost-effective, and environmentally conscious marine infrastructure [281].

## 4. Applications of FRCs in Marine Decks and Underwater Structures

### 4.1. Naval and Commercial Ships

The shipbuilding industry has undergone a significant transformation with the increasing adoption of FRCs, driven by the need for lightweight, fuel-efficient, and durable marine structures [1,4,41,165]. Unlike traditional materials such as steel and aluminum, FRCs offer exceptional resistance to electrochemical corrosion, reducing maintenance costs and extending the service life of vessels [4,152]. Their design flexibility allows for complex geometries, enabling optimized hydrodynamic performance and structural efficiency [188,194]. These advantages have made FRCs indispensable in modern naval and commercial shipbuilding, particularly for hulls, superstructures, decks, and propulsion systems [1,5].

GFRPs dominate the construction of small to medium-sized vessels, accounting for over 90% of recreational boats and patrol crafts [1,61,295,296,297]. The widespread use of GFRP stems from its cost-effectiveness, ease of fabrication, and excellent corrosion resistance [96,100]. For smaller hulls, a combination of chop-strand mat (CSM) and polyester resin is commonly employed due to its affordability and sufficient mechanical properties [25,298]. In contrast, naval vessels often utilize woven roving reinforcements with vinyl ester resins to enhance impact resistance and durability in demanding operational environments [299].

**Figure 10:**
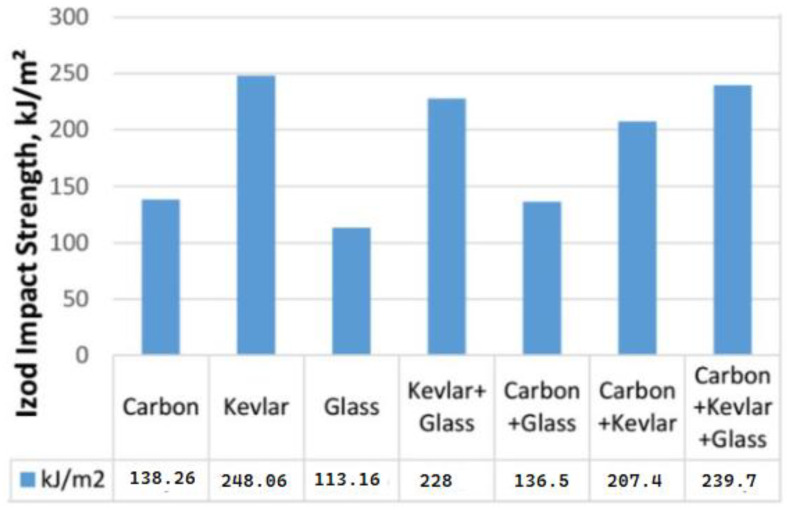
Impact strength properties Change with hybrid FRCs systems, Reproduced from [300], Polish Academy of Sciences, 2017.

CFRP has emerged as a superior alternative for high-performance applications, particularly in naval ships such as the Combat (military) Ships [1]. The integration of CFRP in hull construction results in a 30–40% reduction in weight compared to traditional steel structures, significantly improving speed and maneuverability [15,116,152]. Additionally, hybrid composite designs, such as carbon-aramid laminates, are increasingly used in naval superstructures to enhance blast resistance and structural integrity under extreme conditions [89,137]. These hybrid systems combine the high stiffness of carbon fibers with the exceptional impact absorption of aramid fibers, making them ideal for military vessels where survivability is paramount [15]. As shown in Figure 10, the impact strength properties demonstrate significant improvement when using hybrid FRC systems compared to single-fiber configurations [300].

The use of sandwich composites in marine decks has become a standard practice due to their superior stiffness-to-weight ratio and thermal insulation properties [24,259]. A typical sandwich panel consists of a lightweight PVC foam core bonded to GFRP skins, providing a flexural modulus (E) in the range of 10–15 GPa while maintaining low thermal conductivity [110,301]. This design is particularly advantageous for passenger ships, where comfort and energy efficiency are critical [110,301].

Bulkheads in naval vessels often incorporate aramid FRCs, such as Kevlar-epoxy laminates, to withstand ballistic impacts and explosive loads [1,2]. These materials exhibit exceptional energy absorption capabilities, with impact resistance ranging from 50 to 100 kJ/m^2^, making them indispensable in warships and aircraft carriers [1,5]. The ability of aramid composites to dissipate energy through fiber deformation and delamination ensures the structural integrity of critical compartments, enhancing crew safety during combat scenarios [85,89]. Table 10 presents the applications of fiber-reinforced composites (FRCs) in shipbuilding.

**Figure 11:**
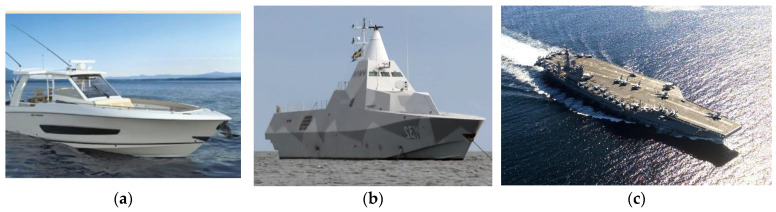
(**a**) Outrage 420 (GFRP), Adapted from [303], Composites Lab., (**b**) Visby-class corvette (CFRP) Adapted from [304], Saab Group. (**c**) Nimitz-class aircraft carrier (Kevlar), Adapted from [305], Wikipedia.

Figure 11 shows real examples of FRC use in shipbuilding: (a) The Outrage 420 is a recreational boat made with GFRP, offering durability, low maintenance, and corrosion resistance [303]. (b) The Visby-class corvette, a naval ship, uses CFRP to reduce weight and radar signature while improving speed and efficiency [304]. (c) The Nimitz-class aircraft carrier uses Kevlar composites in bulkheads to resist blasts and impacts, improving safety in combat [305]. These cases demonstrate how various FRCs enhance performance and protection in modern marine vessels.

### 4.2. Offshore Oil and Gas Platforms

The offshore oil and gas industry has witnessed a paradigm shift in material selection with FRCs increasingly replacing conventional steel components in critical infrastructure [1,2,3,165]. This transition is primarily driven by the exceptional corrosion resistance and significant weight reduction offered by composite materials, which translate to extended service life and reduced maintenance costs in harsh marine environments [2,165]. The unique properties of FRCs make them particularly suitable for walkways, helidecks, and subsea systems where performance and safety are paramount considerations [1,2]. Table 11 presents the applications of fiber-reinforced composites (FRCs) in offshore platforms.

GFRP gratings have become the material of choice for offshore platform walkways, offering a remarkable 50% weight reduction compared to traditional steel gratings while maintaining comparable structural integrity [1,100]. These composite gratings feature specially engineered slip-resistant surfaces with a roughness average (R_a_), ensuring worker safety even in wet or oily conditions [306]. The inherent chemical inertness of GFRP makes it resistant to the corrosive effects of seawater, hydrocarbons, and cleaning chemicals commonly encountered in offshore environments [217,306].

The implementation of basalt-fiber reinforced sandwich panels in helideck construction represents a significant advancement in offshore safety technology [2,307]. These innovative composite structures demonstrate exceptional fire performance, maintaining structural integrity for over 60 min when exposed to temperatures reaching 800 °C [308]. The thermal stability of basalt fibers, combined with phenolic resin systems, creates a protective barrier that is crucial for safety on offshore platforms [132]. Another breakthrough in deck protection comes from self-healing coating systems incorporating microencapsulated dicyclopentadiene (DCPD), which autonomously repair microcracks through a polymerization mechanism triggered by mechanical damage [80,309].

**Table 11:** FRC applications in offshore platforms.

Material	Application	Key Advantage	Examples	Ref.
GFRP/Vinyl Ester CFRP/Epoxy	Walkways	Slip resistance, chemical inertness	Stairways and walkways e.g., Figure 12a	[1,306]
GFRP	Helidecks	Fire resistance, high strength	Helidecks on oil platforms	[310]
CFRP/Thermoplastic CFRP/Epoxy	Risers	Fatigue resistance, buoyancy	Deep-water production risers e.g., Figure 12b	[1,3,8,22]

**Figure 12:**
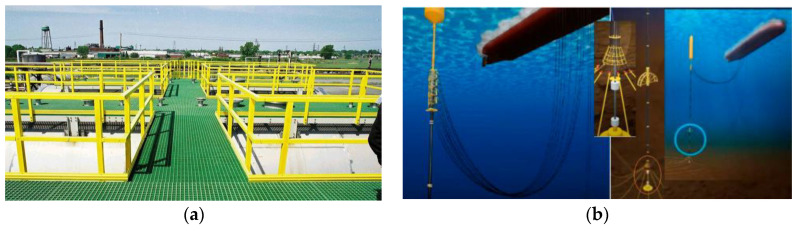
(**a**) Walkaways (GFRP), Adapted from [311], cofiberial. (**b**) Deep-water production risers (CFRP), Reproduced from [8], MDPI, 2022.

The development of thermoplastic composite pipes (TCPs) has revolutionized subsea infrastructure by offering a flexible, corrosion-free alternative to conventional steel risers. These advanced composite pipes combine the durability of carbon fiber reinforcement with the chemical resistance of high-performance thermoplastics, resulting in systems that are immune to the galvanic corrosion and hydrogen embrittlement that plague metallic alternatives [2,8,310,312]. For applications requiring additional structural performance, hybrid riser systems combining steel with composite materials have emerged as an optimal solution, balancing the cost-effectiveness of steel with the weight savings and corrosion resistance of composites. These hybrid systems are particularly valuable in deep-water installations where both performance and economic considerations are critical [8,313].

North Sea oil platforms like Forties Alpha and Brent Bravo use GRP gratings and walkaways due to their corrosion resistance, low weight, and slip resistance. These properties enhance safety and reduce maintenance, making them ideal for offshore walkways exposed to harsh marine conditions [314]. In subsea systems, Airborne Oil & Gas has successfully installed TCP risers for Shell and Total Energies in deep-water fields, demonstrating reduced installation time and lifecycle costs [22,315]. These cases showcase the operational benefits and growing acceptance of FRC technologies in offshore oil and gas applications.

### 4.3. Submarines and Underwater Structures

The application of FRCs in submarine and deep-sea structures has revolutionized underwater engineering, enabling unprecedented performance in extreme pressure environments [2,12,13,316]. Table 12 presents the applications of fiber-reinforced composites (FRCs) in underwater systems.

CFRP pressure hulls represent a breakthrough in submarine design, with modern vessels like Trident and uncrewed submarines demonstrating collapse depths [1,2,12]. These advanced composite structures achieve their remarkable performance through optimized fiber architecture and resin systems that maintain structural integrity under immense hydrostatic pressures [2,61]. The anisotropic nature of CFRP allows engineers to tailor material properties specifically for compressive loading conditions encountered in deep diving operations, resulting in weight reductions of 20–30% compared to equivalent steel hulls while maintaining equivalent or superior strength characteristics [23,317].

Sonar dome construction has similarly benefited from composite material innovation. Glass-epoxy composites have become the material of choice for these critical acoustic components due to their exceptional acoustic transparency, with transmission losses measuring less than 1 dB across operational frequency ranges [1,217,227].

FRCs, especially CFRP and GFRP, are increasingly used in underwater buoyancy modules for applications such as Remotely Operated Vehicles (ROV) and Autonomous Underwater Vehicles (AUV) [23,318,319]. These materials offer excellent strength-to-weight ratios, corrosion resistance, and fatigue performance [23,317]. FRC-based sandwich structures with foam or polymer cores ensure uniform pressure distribution and structural integrity under extreme depths [316,319]. Their integration improves buoyancy control, enhances operational safety, and reduces weight, making them ideal for deep-sea marine systems [316,317,318].

**Table 12:** FRC applications in underwater systems.

Material	Application	Key Advantages	Example	Ref
High-modulus CFRP	Pressure hulls	Collapse depths, 20–30% weight savings	Trident-class, unmanned subs	[2,12,320]
GFRC/epoxy	Sonar domes	<1 dB transmission loss	Naval sonar domes	[1,320]
CFRP GFRP	Buoyancy modules	Strength-to-weight ratios, corrosion resistance	ROVs, AUVs e.g., Figure 13a,b	[23,318,319]

**Figure 13:**
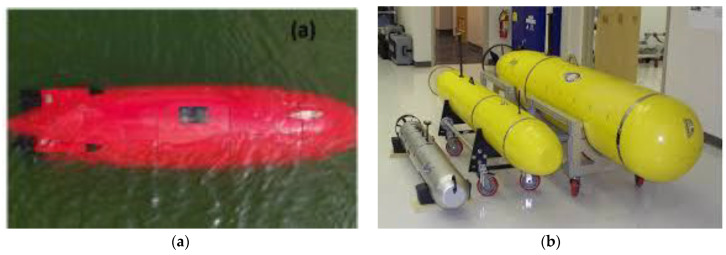
(**a**) DIVE-LC AUV, Reproduced from [316], MDPI, 2024. (**b**) The Bluefin-21 (large) AUV, Reproduced from [322], MDPI, 2022.

Figure 13 presents the DIVE-LC and Bluefin-21 AUVs, both using fiber-reinforced composites for lightweight, corrosion-resistant hulls. These materials improve durability and performance, enabling deep-sea exploration and military missions with enhanced endurance and reliability under extreme underwater conditions [316,321,322].

### 4.4. Floating Infrastructure and Sustainability Outlook

**Figure 14:**
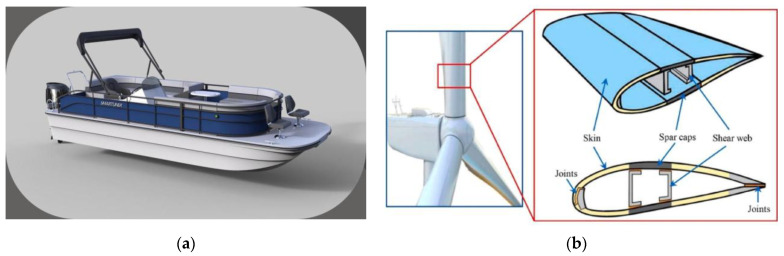
(**a**) GFRP Pontoon, Adapted from [326], Smartliner Boat. (**b**) GFRP wind turbine, Adapted from [24], Elsevier, 2024.

The marine industry has witnessed transformative advancements in floating infrastructure through the implementation of GFRP pontoon systems [2,24,224,323]. These modular composite structures have revolutionized military bridging applications, where rapid deployment capabilities are critical for operational success [324]. The inherent corrosion resistance of GFRP materials eliminates the need for protective coatings required by traditional steel pontoons. In contrast, their lightweight properties (typically 60–70% lighter than equivalent steel structures) enable faster assembly and reduced logistical requirements [2,312,325]. Figure 14a visually illustrates a GFRP pontoon example, emphasizing the lightweight and modular nature of these systems.

The transition to renewable energy has driven significant innovation in offshore wind turbine platform design, where GFRP composites are increasingly replacing conventional steel structures. Composite platforms offer a nearly 50% reduction in weight compared to steel alternatives, dramatically decreasing installation costs and enabling the use of smaller installation vessels [24,165,307,323]. As shown in Figure 14b, GFRP wind turbines highlight the growing adoption of composites in large-scale renewable energy infrastructure.

In high-performance applications, CFRP composites are being explored for their superior stiffness-to-weight ratio and fatigue resistance, particularly in structural components such as floating foundation tension members, blades, and dynamic power cables [2]. Although more expensive than GFRPs, CFRPs deliver exceptional mechanical performance under cyclic ocean loading and exhibit minimal creep deformation, making them ideal for deep-water floating wind platforms [1,316]. Their low thermal expansion also supports dimensional stability in varying temperatures, which is crucial for long-term offshore durability [74,316].

## 5. Challenges and Limitations of FRCs in Marine Deck Applications

FRCs have emerged as promising materials for marine deck structures due to their high strength-to-weight ratio, corrosion resistance, and design flexibility [8,85]. However, their widespread adoption faces significant challenges related to long-term durability, manufacturing complexities, regulatory hurdles, and economic considerations.

### 5.1. Long-Term Durability Concerns

The marine environment presents extreme conditions that challenge the durability of FRCs. Seawater exposure leads to moisture absorption, which can cause matrix plasticization, fiber-matrix debonding, and hydrolysis of polymer networks. Studies show that epoxy-based glass fiber composites may lose 20–30% of their flexural strength after prolonged seawater immersion due to these degradation mechanisms [3,20,200]. The moisture absorption typically follows Fickian diffusion initially but becomes more complex as microcracks develop, allowing accelerated fluid penetration [3,260].

CFRP composites demonstrate better seawater resistance but face unique challenges. When coupled with metals, they can initiate galvanic corrosion, significantly accelerating degradation [272,273]. Their electrical conductivity also increases susceptibility to lightning strikes, requiring additional protective measures [8,152]. Thermoplastic matrices like PEEK show superior performance, with studies reporting less than 0.5% moisture absorption after 1000 h of immersion and minimal property changes [25,27].

Basalt fiber composites have gained attention as environmentally friendly alternatives, but their performance in alkaline seawater remains problematic. Research indicates significant strength reduction (15–25%) due to chemical attack on the fibers [21,271]. Hybrid systems combining basalt with synthetic fibers show promise in balancing cost and performance [214].

Fatigue behavior in marine conditions presents another critical concern. The combination of cyclic loading and seawater exposure can reduce fatigue life by up to 40% compared to dry conditions, primarily through accelerated matrix cracking and delamination [215,247]. This effect is particularly pronounced at stress concentrations and in woven fabric composites. Table 13 presents the seawater degradation effects on marine composites, detailing the material system, key degradation mechanisms, and resulting property reduction.

Recent advancements aim to address these durability challenges. Nano-modified matrices incorporating silica or CNTs demonstrate improved barrier properties [157,237]. In addition, the use of multifunctional nanomaterials has been shown to enhance mechanical strength, thermal stability, and resistance to environmental degradation [144,154]. Hybrid fiber architectures optimize performance while mitigating individual material limitations [307]. Advanced coating systems, including graphene-based and self-healing variants, show promise for long-term protection [147,327].

Beyond laboratory findings, real-world deployment of FRCs must account for classification society standards such as Bureau Veritas and ABS rules, which set safety, fire, and durability requirements for marine decks [328]. Field evidence shows significant property loss in service: epoxy/glass laminates have exhibited tensile strength reductions up to 49% after 12 months of seawater exposure, with biofouling and matrix degradation as key failure mechanisms [270,329,330]. Such case studies highlight the gap between accelerated aging tests and actual service conditions. Incorporating Life Cycle Assessment (LCA) into design decisions helps balance mechanical performance, recyclability, and environmental footprint [110,328].

LCA studies reveal that material choice, e.g., thermoset vs. thermoplastic vs. natural fiber, can significantly alter end-of-life impacts and compliance with circular economy goals [110,328]. Addressing these factors through predictive durability models, harmonized testing standards, and sustainable material selection is critical for reliable, certifiable, and environmentally responsible FRC adoption in marine deck applications [110,270,328,329].

### 5.2. Manufacturing and Repairability Challenges

The production of large marine deck components from FRCs involves complex manufacturing processes with specific limitations. VARTM is widely used but requires precise control of resin viscosity and infusion parameters. Improper processing can lead to dry spots and resin-rich areas, reducing mechanical properties by up to 20% [18,171]. Recent developments in low-viscosity bio-based resins have improved processability while maintaining performance [39].

AFP offers advantages for complex geometries but faces adoption barriers. While reducing material waste by 30% compared to hand layups, the high equipment costs (exceeding USD 1 million) and specialized operator requirements limit its use in smaller shipyards [19]. The process is primarily optimized for aerospace thermoset prepregs, requiring adaptation for marine applications [25].

Thermoplastic composites present unique manufacturing challenges. The high melting temperatures of engineering thermoplastics like PEEK (∼343 °C) demand specialized equipment and significant energy input [177]. Achieving uniform consolidation without thermal degradation remains challenging, particularly for thick sections.

Repairability differs significantly between material systems. Thermoset composites require extensive surface preparation and controlled curing conditions, often necessitating dry-docking. Thermoplastics allow welding repairs but need specialized equipment [178]. Moisture contamination and uneven curing in field conditions complicate in-situ repairs. Table 14 compares marine composite manufacturing methods, outlining each method’s advantages and limitations.

Emerging solutions include in-situ polymerization techniques to reduce processing temperatures and digital twin technologies for process optimization [19,80]. Modular design approaches using prefabricated components are gaining traction to simplify both manufacturing and repairs [331].

### 5.3. Regulatory and Certification Issues

Marine composites must comply with stringent safety and environmental regulations, creating significant adoption barriers. Fire safety presents particular challenges, as traditional composites often fail to meet smoke and toxicity requirements [280]. Flame-retardant additives like ammonium polyphosphate improve performance but increase costs and affect processing [281].

Certification processes are lengthy and expensive, often taking up to two years and costing hundreds of thousands of dollars [196,256]. The lack of standardized accelerated aging protocols complicates material qualification, as different classification societies may require different testing regimens [307]. Environmental regulations increasingly restrict hazardous chemicals used in composite production. Styrene emissions from polyester resins have driven the development of low-styrene alternatives [60]. Concerns about micro plastic pollution are prompting research into more stable resin systems and natural fibers [90].

The variety of material systems and manufacturing methods further complicates certification. Each combination may require separate approval, creating a significant burden. Some progress has been made in “generic” material qualifications; however, their adoption remains limited [15,22]. Industry efforts to address these challenges include harmonizing standards across classification societies and using computational modeling to predict long-term performance [22]. Digital documentation systems, including blockchain-based traceability, may streamline certification while maintaining quality control [331].

While existing standards such as ASTM D3039 (tensile testing), D7264 (flexural properties), and ISO 13003 (fatigue) provide a foundation for composite material qualification, they were not explicitly designed for the harsh, variable conditions of marine environments [207,245]. Classification societies like DNV and ABS offer marine-focused guidelines (e.g., DNV-GL for composites and ABS standards for FRP hulls), but critical gaps remain [22,313]. Notably, there is no consensus on accelerated aging protocols that accurately simulate long-term seawater exposure or cyclic loading in submerged conditions [332]. Hybrid systems—such as FRP-steel interfaces—lack standardized testing methods for galvanic corrosion [302]. At the same time, high-performance thermoplastics are often evaluated using aerospace-derived criteria that may not address marine-specific degradation mechanisms such as biofouling or hydrothermal aging [333].

To bridge these gaps, we advocate for a structured, three-tiered certification approach: (1) baseline mechanical characterization using existing ASTM/ISO protocols, (2) modified accelerated aging tests incorporating combined salinity, temperature, and hydrodynamic stressors, and (3) field validation via structural health monitoring (SHM) systems (e.g., Fiber Bragg gratings or CNT-based sensors) to track real-world performance. Collaborative efforts between regulatory bodies (IMO, DNV, ABS), industry stakeholders, and researchers will be essential to develop unified, marine-specific standards that balance safety, durability, and innovation for next-generation FRC applications.

### 5.4. Economic and Sustainability Considerations

**Figure 15:**
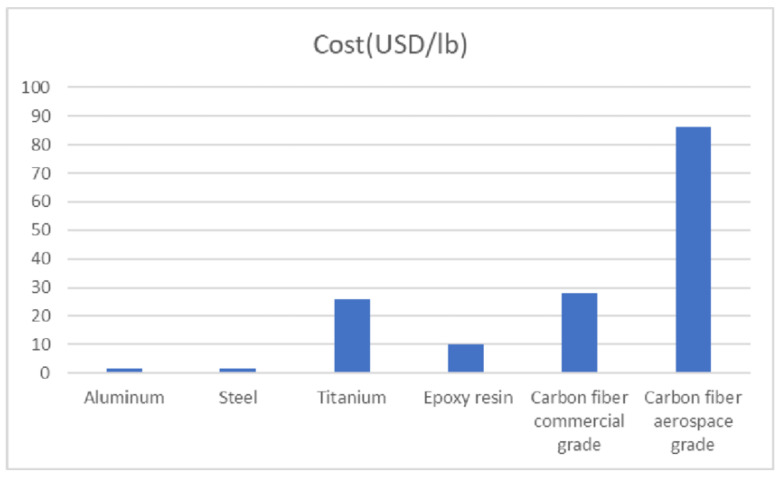
Steel, Aluminum, Titanium: Initial cost comparison with CFRP, Reproduced from [335], Research Square, 2021.

The high initial cost of advanced FRCs remains a primary adoption barrier. Carbon fiber (∼USD 15–20/kg) makes CFRP components significantly more expensive than steel equivalents (Figure 15) [306]. While lifecycle cost analyses show potential savings through reduced maintenance, the higher upfront investment often deters ship owners [296,334].

Sustainability concerns are increasingly important in material selection. Traditional thermoset composites are difficult to recycle, with most end-of-life components ending up in landfills. Only about 30% of GFRP waste is currently recycled, primarily as filler material [296]. The cross-linked nature of thermosets prevents simple reprocessing, requiring energy-intensive chemical recycling methods [81].

Thermoplastic composites offer better recyclability but face other sustainability challenges. The high energy requirements for processing engineering thermoplastics result in significant carbon footprints [25,334]. Bio-based resins provide more sustainable alternatives but often compromise mechanical performance [39,334]. Natural fiber composites show potential but face durability limitations in marine environments. Properly treated natural fibers can achieve satisfactory performance in some applications, but long-term structural use remains unproven [86]. Circular economic approaches are being developed to improve sustainability. Chemical recycling methods can recover fibers while preserving up to 90% of their strength, though the processes remain energy-intensive [43,66]. Alternative strategies include designing for disassembly and developing depolymerizable matrix systems [81].

Table 15 presents mechanical, economic, and environmental impact data for various FRP composites, highlighting trade-offs between strength, cost per functional unit (FU), and sustainability indicators. Carbon fiber epoxy shows high strength with relatively low environmental impact, while natural fiber composites, though sustainable, have lower performance and higher impact per FU [334].

From an economic perspective, production scalability remains challenging. While other industries have advanced composite manufacturing, marine applications require different material forms and processes. The lack of standardized, high-volume production techniques keeps costs elevated compared to metal fabrication [306]. Emerging business models, such as component leasing or take-back programs, aim to improve viability. These approaches require significant changes to traditional shipbuilding practices and supply chains [331]. As the industry addresses these challenges, FRCs are poised to play an increasingly important role in marine deck applications.

## 6. Future Prospects and Research Directions

Recent innovations in marine FRCs include self-healing systems, advanced protective coatings, and sustainable bio-based materials. These advancements aim to enhance durability, resist environmental degradation, and support eco-friendly manufacturing, addressing current limitations while meeting the growing demand for high-performance and sustainable marine engineering solutions [34,66].

### 6.1. Self-Healing and Smart Composites for Structural Longevity

The integration of self-healing capabilities into FRCs represents a transformative advancement in enhancing the durability of marine structures. In harsh marine environments characterized by saltwater exposure, mechanical fatigue, and biological fouling, traditional composites are prone to microcracking and delamination [83,309,327]. Self-healing systems aim to restore mechanical integrity after damage autonomously, extending service life and reducing maintenance demands [83,165,336]. Prominent mechanisms include microencapsulated healing agents, vascular networks, and reversible covalent bonding, with microencapsulation gaining popularity due to its compatibility with standard manufacturing [309,327,336,337]. However, most systems lack validation under realistic marine conditions [1]. Saltwater ingress, cyclic loading, and marine biofouling can hinder healing performance, and many systems are ineffective under high-cycle fatigue common in marine applications [246,277]. Marine-adapted healing chemistries, resilient to moisture and biological degradation, are critical research priorities [256,309].

Concurrently, smart FRCs with embedded sensing systems are revolutionizing structural health monitoring (SHM). Fiber Bragg gratings (FBGs) and CNT networks enable real-time detection of strain, fatigue, and delamination. These technologies support predictive maintenance strategies, reducing catastrophic failure risks [24,66,331,338]. However, implementation challenges persist, including corrosion of metallic sensors in saline environments and limited energy autonomy. Solutions under exploration include corrosion-resistant sensors and energy-harvesting technologies like piezoelectric and triboelectric coatings [306,338,339].

Future innovations should focus on multifunctional composites that integrate self-healing, SHM, and energy harvesting [66,306,338]. Smart coatings that serve dual functions in monitoring and power generation offer a promising long-life and low-maintenance marine composite structures tailored for demanding oceanic conditions [35,338].

### 6.2. Advanced Coating Technologies for Durability and Biofouling Resistance

Advanced coatings are vital for improving the durability and biofouling resistance of FRCs in marine environments. Marine exposure subjects materials to saltwater corrosion, UV radiation, biological fouling, and mechanical wear. Recent innovations leverage nanotechnology, functional materials, and eco-friendly chemistries to enhance surface resilience and meet global environmental standards [35,36,84,277].

Superhydrophobic coatings, such as fluoropolymer–silica hybrids, inhibit biofouling by resisting microbial adhesion and enabling self-cleaning, achieving up to 60% reduction in fouling in lab simulations [70,277,340]. These coatings can reduce drag, improve fuel efficiency, and mitigate the spread of invasive species. Meanwhile, biocidal nanocomposite coatings using nanoparticles like Cu_2_O and Ag offer targeted antimicrobial action while adhering to IMO toxicity guidelines [35,340,341].

Multifunctional coatings are also being developed to combine corrosion resistance, flame retardancy, and self-healing capabilities. For example, nanostructured matrices incorporating cerium oxide, graphene oxide, and healing agents offer multi-modal protection in a single layer [34,160,341]. An emerging frontier lies in smart, stimuli-responsive coatings that adapt to environmental cues (e.g., pH, temperature, salinity). These systems can modulate hydrophobicity or release biocides on demand, reducing environmental impact while enhancing performance. Nevertheless, their marine-specific application is nascent and requires extensive field validation [327].

### 6.3. Bio-Based and Sustainable Composite Systems

Growing environmental regulations and the marine industry’s push to reduce carbon emissions are accelerating the development of sustainable FRCs [4,128,165]. Bio-based and recyclable systems offer compelling environmental advantages while targeting mechanical performance suitable for marine use [34,165,342].

Bio-resin matrices from renewable feedstocks like lignin have achieved more mechanical strength than traditional epoxies. However, their slower curing kinetics and reduced crosslinking under humid, ambient conditions pose processing challenges, requiring chemical modification for marine viability [39,80]. In parallel, natural fibers such as flax, jute, hemp, and basalt are gaining traction for their biodegradability and low embodied energy [15,26,89]. However, their hydrophilicity can lead to moisture-induced degradation [165]. Strategies like silane treatments, nanocellulose fibers, and fiber hybridization improve interfacial bonding and water resistance [34,91,165]. Basalt fibers, offering corrosion resistance and strength, are particularly promising for semi-structural components [122,132,343].

Recyclable thermoplastic matrices such as Elium™, PEEK, and PA are another key innovation [15,25]. These enable closed-loop recycling through pyrolysis or enzymatic degradation while providing enhanced impact resistance. Elium™, in particular, is compatible with conventional infusion processes, aiding adoption [25,66,110,340].

Despite advances, cost barriers and the absence of standardized recycling protocols limit industrial deployment. Bio-resins and thermoplastics remain more expensive than conventional systems, and end-of-life strategies tailored to marine composites are underdeveloped [37,39].

Initiatives like the EU Horizon 2020 ECO-BOAT project are pioneering sustainable marine prototypes, emphasizing full lifecycle integration. Moving forward, cross-sector collaboration, international standardization, and policy incentives are crucial to scale sustainable marine composite technologies [241].

### 6.4. Hybrid Composite Systems for Marine Applications

The development of hybrid composite systems marks a significant advancement in marine structural engineering, combining the strengths of different materials to overcome limitations of traditional composites [344,345]. FRP-reinforced concrete systems demonstrate particular promise for marine infrastructure, offering superior corrosion resistance and reduced maintenance compared to steel-reinforced concrete. It also allows for a material cost reduction of over 60% compared to the steel platform design [344,346]. Recent studies show these systems exhibit less deterioration in coastal zones over more extended periods of exposure [345]. Steel-FRP hybrids are gaining traction for hull construction, where they combine steel’s impact resistance with FRP’s weight savings and corrosion protection [302]. These hybrid systems address critical challenges in marine environments while enabling innovative structural designs. Current research focuses on optimizing interfacial bonding, fatigue performance under cyclic wave loading, and developing standardized testing protocols. The integration of hybrid composites is transforming marine construction by providing solutions that balance durability, weight reduction, and lifecycle costs [344,347]. Table 16 presents the performance characteristics of marine hybrid composites.

## 7. Conclusions

### 7.1. Summary of Key Findings

FRCs have demonstrated a transformative potential in marine decks and underwater structures, outperforming traditional materials (steel, aluminum) in weight savings (20–40%), corrosion resistance, and design flexibility. Thermosets (epoxy, vinyl ester) dominate current applications due to their established performance, while thermoplastics (PEEK, PEKK) and bio-based resins emerge as sustainable alternatives with superior recyclability and hydrothermal stability. Key challenges persist in long-term durability (e.g., moisture absorption, fatigue degradation), manufacturing scalability, and regulatory compliance, necessitating advanced solutions like self-healing systems and nanomaterial-enhanced matrices.

### 7.2. Potential of FRCs in Marine Decks

FRCs are poised to redefine marine engineering, enabling lightweight, fuel-efficient vessels, and resilient offshore infrastructure. Innovations in smart composites (e.g., embedded sensors, stimuli-responsive coatings) and circular economy models (recyclable thermoplastics, bio-resins) align with global sustainability goals. The integration of hybrid materials (e.g., carbon–basalt hybrids) and AI-driven predictive maintenance could further enhance reliability in extreme environments.

### 7.3. Recommendations

- Industry Adoption: Standardize accelerated aging protocols and certification pathways for novel FRCs to streamline regulatory approval.
- Research Focus: Prioritize in situ performance validation of self-healing systems, scalable recycling methods, and multifunctional coatings combining antifouling/fire retardancy.
- Policy and Collaboration: Foster cross-sector partnerships to commercialize bio-based composites and digital twins for lifecycle management.

By addressing these gaps, FRCs can achieve widespread adoption, offering safer, greener, and cost-effective solutions for next-generation marine infrastructure.

## Figures and Tables

**Table 1:** Properties of common marine-grade thermoset resins.

Material	Flexural		Tensile Strength (MPa)	Compression Strength (MPa)	Chemical	Corrosion	Cost	Applications	References
	Strength (MPa)	Modulus (GPa)			Resistance				
Epoxy	85–120	3.0–4.5	60–90	100–140	Excellent (RILEM PC 12, RILEM PCM8) ^1^	Very good (Real Land Composite) ^2^	More expensive than polyester and vinyl ester	Hulls, structural components, high-stress areas, fuel tanks, and bilges	[4,5,49,50,51,52,53]
Polyester	50–90	2.0–3.5	40–75	80–110	Good (Varies) (RILEM PC 12, RILEM PCM8) ^1^	Moderate (Canadian Composite Structures, INC) ^3^	Less Expensive than steel	Non-critical parts	[49,50,54,55,56,57]
Vinyl ester	80–110	2.8–4.0	70–95	90–130	Very Good (Real Land Composite) ^2^	Very Good (Canadian Composite Structures, INC) ^3^	More expensive than polyester	Hulls, tanks, and components exposed to saltwater	[4,5,49,57,58,59,60]

RILEM PC 12—“Method of test for chemical resistance of polymer concrete”, RILEM PCM8—“Method of test for flexural strength and deflection of polymer-modified mortar” ^1^, Changzhou Real Land Composite Material Technology Co., Changzhou, China ^2^, and Canadian Composite Structures, Inc., Woodstock, ON, Canada, which is a global manufacturer ^3^.

**Table 2:** Properties of common marine-grade thermoplastic resins.

Material	Flexural		Tensile Strength (MPa)	Compression Strength (MPa)	Chemical	Corrosion	Cost-Effectiveness	Best Uses in Marine Applications	References
	Strength (MPa)	Modulus (GPa)			Resistance				
					* This Comparison is Based Solely on the Materials Listed in the Table				
PE	10–30	0.8–1.5	10–40	15–50	Excellent	Excellent	Low	Buoyancy aids, liners, and pipes.	[69,75,76]
PP	20–40	1.0–2.0	30–50	20–60	Excellent	Excellent	Low	Ropes, nets, liners, and lightweight components.	[69,74,75,76]
PEEK	150–170	3.5–4.5	90–120	150–180	Excellent	Excellent	Very High	High-performance components: Bearings, seals, propellers, and underwater connectors.	[70,74,75]
PEKK	140–160	3.0–4.0	85–110	140–170	Excellent	Excellent	Very High	High-temperature and chemical-resistant parts: Engine components, pump housings, and structural parts.	[11,70,75]
Elium™	80–100	2.5–3.5	60–80	70–100	Good	Good	High	Lightweight composite structures: Hulls, panels, and repair materials.	[38,42,63]
PA	50–120	2.0–3.0	60–100	80–130	Good to Excellent	Good	Moderate	Gears, bushings, and structural parts.	[68,75,76]
PLA	50–70	3.0–4.0	40–65	50–80	Moderate	Moderate	Low	Biodegradable marine products: Temporary fixtures.	[12,64,69]
PHA	20–40	1.0–2.0	20–45	30–60	Moderate	Moderate	Moderate	Biodegradable marine products: Fishing nets, packaging.	[67,77,78]

* The comparison presented here is based only on the materials listed in the table. The results may vary if other composites or materials are considered.

**Table 3:** Comparative analysis: Thermoset vs. thermoplastic.

Aspect	Thermoset Composites	Thermoplastic Composites	References
Manufacturing Complexity	Lower viscosity; easier processing at moderate temps	High viscosity; requires high temps and specialized equipment	[5,11,12,29,42,79,80,81,82]
Recyclability	Non-recyclable due to cross-linked matrix	Recyclable and reparable due to the thermoplastic nature	
Interfacial Bonding	Covalent/secondary interactions	Challenging in marine conditions	
Mechanical Performance	Well-established strong adhesion and rigidity	Good impact resistance; bonding and durability under marine conditions need improvement	
Cost	Generally lower material and processing costs	Higher material cost and processing complexity	
Environmental Impact	Less sustainable; typically petroleum-based	More sustainable; potential for bio-based and recycled materials	
Shelf Life	Limited shelf life due to curing requirements	Infinite shelf life; can be remelted and reshaped	

**Table 4:** Summary of fiber types.

Fiber Type	Common Resin Systems	Bonding/Compatibility Notes	Recommended Marine Use/Environment	References
Glass Fiber	Polyester, vinyl ester, and epoxy; also used with thermoplastics like PP, PA, and PEEK	E-glass provides good mechanical properties, electrical insulation, and moisture resistance; S-glass has higher tensile strength and stiffness—excellent compatibility with thermosets, especially epoxy. Thermoplastics offer recyclability and high impact resistance.	General marine structures such as small boats’ hulls, decks, and bulkheads; corrosion-prone environments. S-glass for high-performance components.	[1,61,74,93,94,95,96,98]
Carbon Fiber	Epoxy (preferred), vinyl ester, and polyester; also with PEEK, PA, and thermoplastics	Epoxy provides strong adhesion and low moisture absorption; vinyl ester offers good water resistance; polyester is lower cost but less durable. Thermoplastics (PEEK, PA) provide high toughness, fast processing, and recyclability.	High-performance marine structures such as naval vessels, hydrofoils, and submersibles are ideal where a high strength-to-weight ratio and low moisture uptake are critical.	[1,8,16,107,112,113]
Basalt Fiber	Epoxy, vinyl ester, and polyester; thermoplastics like PA, PP, and PEEK	Similarly to the processing of glass fiber, epoxy offers the best performance. Vinyl ester provides good water resistance, and polyester is suitable for cost-sensitive applications. Thermoplastics enhance recyclability and processing speed.	Hulls, offshore rigs, and pipelines in corrosive or high-heat marine environments; increasing interest in sustainable applications.	[121,122,123,124,125,127,132]
Aramid Fiber	Epoxy, polyester, and vinyl ester (less common); PEEK, PA (thermoplastics)	Surface treatments and coatings are needed for achieving strong bonding. Para-aramids provide structural strength, while meta-aramids are known for thermal resistance. Thermoplastics offer recyclability and toughness. UV protection is required for exposed areas.	Impact- and abrasion-resistant marine zones; reinforcement of hulls, safety nets, and protective barriers. Used in demanding safety or performance applications.	[133,134,135,137,138,139,140]

**Table 5:** Commonly used fibers in composites. Reproduced from [132], MDPI, 2022.

Fiber Type	Fiber Diameter (µm)	Density (g/cm^3^)	Tensile Strength (MPa)	Modulus of Elasticity (GPa)	Elongation at Break (%)	Price (USD/kg)
Basalt	9–23	2.8–3.0	3000–4840	79.3–93.1	3.1	2.5–3.5
E-glass	9–13	2.5–2.6	3100–3800	72.5–75.5	4.7	0.75–1.2
S-glass	9–13	2.46–2.5	4590–4830	88–91	5.6	5–7
Carbon	4–7.5	1.75–1.9	3500–6000	230–600	1.5–2.0	30
Aramid	5–18	1.44	2900–3400	70–112	2.8–3.6	25

**Table 6:** Critical comparison of fiber types for marine applications.

Fiber Type	Tensile Strength (MPa)	Moisture Absorption	Corrosion Resistance	Fatigue Performance	Cost (USD/kg)	Sustainability	Best Marine Uses	References
E-glass	Moderate 3100–3800	High (0.5–1.0%)	Excellent	Moderate (30–50% reduction after seawater exposure)	0.75–1.20	Low (energy-intensive production)	Hulls, decks, and non-structural parts	[1,61,74,93,94,95,96,98]
S-glass	High 4590–4830	Moderate (0.3–0.6%)	Excellent	Good (25–40% reduction)	5–7	Low	High-performance naval components	
Carbon	High 3500–6000	Very Low (<0.1%)	Excellent (but galvanic risk)	Excellent (<20% reduction)	15+	Moderate (recyclable but high embodied energy)	Pressure hulls, risers, and hydrofoils	[1,16,112,113] [107]
Basalt	Moderate to High 3000–4840	Moderate (0.2–0.5%)	Excellent	Good (20–35% reduction)	2.5–3.5	High (natural material, low processing energy)	Offshore platforms, fireproof structures	[121,122] [123] [125] [132]
Aramid	Moderate 2900–3400	Low (0.2–0.4%)	Excellent	Exceptional (15–25% reduction)	25	Moderate (difficult to recycle)	Bulletproof panels, impact zones	[133,134] [135] [138]

**Table 7:** Common nanomaterials in the marine industry.

Nanomaterial	Key Properties	Typical Loading	Processing Challenges	Marine Applications	References
Graphene	High strength (130 GPa), conductivity	0.1–1.0 wt%	Dispersion difficulty, high cost	Hulls, sensors	[111,112,115]
					[114,120,121]
CNTs	High aspect ratio (>1000), conductive	0.3–0.8 wt%	Increased resin viscosity	Structural health monitoring	[113,115,120]
					[114,121,124,129]
Nano-clay	Layered structure, flame retardant	2–5 wt%	Exfoliation required	Fireproof bulkheads	[115,116,117]
Nano-silica	High surface area (300 m^2^/g)	1–3 wt%	Agglomeration risk	Deck coatings	[116,118,119]
					[117,123]

**Table 8:** Manufacturing methods.

Manufacturing Method	Compatible Resin Types	Common Resins Used	Nanomaterial Integration	Marine Suitability	Sustainability and Recyclability	References
VARTM	Thermosets	Epoxy (DGEBA), Vinyl Ester, and Polyester	Limited by increased resin viscosity	Hulls, decks, and bulkheads	Moderate (closed mold reduces VOCs; thermosets are not recyclable)	[18,91,167,168,169,172]
RTM	Thermosets	Epoxy, Vinyl Ester	High (nano-silica, CNTs, nano-clay enhancements possible)	High-performance parts (rudders, keels)	Moderate (efficient but limited recyclability due to thermosets)	[18,143,168,170,172]
AFP	Thermosets and Thermoplastics	Epoxy Prepregs, PEEK, and PEKK	Nano-prepregs and tailored nanofiber integration are available	Naval vessels, racing yachts	High (thermoplastics are recyclable; process is energy-intensive)	[19,176,177,178]
ATL	Thermosets and Thermoplastics	Epoxy, PEEK, and Elium™	Nano-enhanced tapes under development	Large panels, hull sections	High (especially with recyclable thermoplastics)	[15,19,177,178]
Pultrusion	Mostly Thermosets; Some Thermoplastics	Polyester, Vinyl Ester, and PP	Surface nano-coatings or filled resins improve bonding	Masts, beams, rails, and pipes	High (with thermoplastics; thermosets still common)	[32,181,182,188,189,190]
Filament Winding	Thermosets and Thermoplastics	Epoxy, PEEK, and Vinyl Ester	Nano-resins enhance burst strength and fatigue resistance	Pressure vessels, tanks, and submersible hulls	High (particularly with thermoplastics)	[166,181,184,185,199]
AM	Primarily Thermoplastics	PLA, PEEK, PEI, PA, and PP	CNTs, graphene, and smart fillers used in research	Prototypes, small or non-structural parts	Moderate (material waste is low, but anisotropy and limited reuse of fiber-reinforced filament)	[188,189,191,193,194]

**Table 9:** Comparative performance of marine FRCs in corrosion and fouling resistance.

Material System	Galvanic Corrosion Risk	Biofouling Rate	Effective Protection Methods	References
CFRP	High (with metals)	Moderate	Glass fiber veils, zinc anodes	[6,271,272,273]
GFRP	None	High	Silicone foul-release coatings	
BFRP	None	Low-Moderate	Nano-ZnO/polyurethane	

**Table 10:** FRC applications in shipbuilding.

Material	Application	Key Advantages	Example	References
GFRP/Polyester	Small boat hulls	Low cost, corrosion resistance	Fishing vessels e.g., Outrage 420 [Figure 11a]	[297,302]
CFRP/Vinyl Ester	Naval ship hulls	High stiffness, weight reduction	Military Ships e.g., Visby-class corvette [Figure 11b]	[1,295,297]
Aramid Hybrid (Kevlar)	Bulkheads	Blast/impact resistance	Aircraft carriers e.g., Nimitz-class aircraft carrier [Figure 11c]	[1,295]

**Table 13:** Seawater degradation effects on marine composites.

Material System	Key Degradation Mechanisms	Property Reduction	References
GFRP (Epoxy)	Matrix swelling, interface degradation	20–30% flexural strength	[200,202]
CFRP (Epoxy)	Galvanic corrosion, interface weakening	10–15% tensile strength	[112,272]
CFRP (PEEK)	Minimal water absorption	<5% property change	[25,27]
Basalt/Epoxy	Alkali attack on fibers	15–25% tensile strength	[21,271]

**Table 14:** Comparison marine composite manufacturing methods.

Method	Advantages	Limitations	References
VARTM	Large parts, low tooling cost	Parameter sensitivity	[18,164]
Resin Infusion	Good wetting	Simple geometries	[167,168]
AFP	Precision, automation	High cost	[19,25]

**Table 15:** Mechanical, Economic, and Environmental Impact Data of Analyzed FRP Composite Materials, Reproduced from [334], Springer, 2021.

Fiber Type	Matrix	Compressive Strength (MPa)	kg/FU	Price (USD /FU)	Human Health (Pt/FU)	Ecosystems (Pt/FU)	Resources (Pt/FU)	Total EI (Pt/FU)
Glass Fiber	Epoxy	600	15.96	485.7	4.29	1.72	3.3	9.32
	Polyester	420	22.59	572.5	5.51	2.69	4.18	12.38
	Vinyl Ester	600	15.08	573.2	3.7	1.55	2.97	8.22
	Thermoplastic	420	22.47	671.3	5.77	2.52	4.13	12.43
Carbon Fiber	Epoxy	1700	4.52	165.1	2.81	1.21	3.37	7.39
	Polyester	1200	6.33	223.7	2.94	1.51	4.06	8.51
	Vinyl Ester	1700	4.21	168.1	2.01	0.98	2.86	5.85
	Thermoplastic	1200	6.29	229.6	3.07	1.47	4.12	8.66
Natural Fiber	Epoxy	150	44.25	942.8	10.35	5.49	8.63	24.47
	Polyester	105	62.38	1106.7	12.35	8.73	10.23	31.32
	Vinyl Ester	150	40.75	1084	8.07	4.81	7.34	20.21
	Thermoplastic	105	61.9	1294.6	13.37	8.11	10.15	31.63
Basalt Fiber	Epoxy	600	16.46	501	3.83	1.68	3.18	8.69
	Polyester	420	23.3	590.6	4.87	2.63	4.01	11.51
	Vinyl Ester	600	15.58	592.2	3.27	1.52	2.85	7.64
	Thermoplastic	420	23.18	692.6	5.12	2.46	3.96	11.55

**Table 16:** Performance characteristics of marine hybrid composites.

System Type	Tensile Strength (MPa)	Corrosion Resistance	Weight Reduction	Service Life (Years)	References
FRP-Reinforced Concrete	80–120	Excellent	20–30%	30+	[302,344,345,347]
Steel–FRP Hybrid	350–500	Good	15–25%	25+	

## Data Availability

The original contributions presented in this study are included in the article. Further inquiries can be directed to the corresponding author.

## Abbreviations

AFP	Automated Fiber Placement
APP	Ammonium Polyphosphate
ATH	Aluminum Trihydroxide
AUV	Autonomous Underwater Vehicle
BFRP	Basalt Fiber-Reinforced Polymer
CFRP	Carbon Fiber-Reinforced Polymer
CNTs	Carbon Nanotubes
CSM	Chop-Strand Mat
DCPD	Dicyclopentadiene
DGEBA	Diglycidyl Ether of Bisphenol A
DIC	Digital Image Correlation
DMA	Dynamic Mechanical Analysis
ECN	Epoxy Cresol Novolac
E-glass	Electrical-Grade Glass Fiber
EIS	Electrochemical Impedance Spectroscopy
EOL	End-of-Life
EPN	Epoxy Phenol Novolac
FBGs	Fiber Bragg Gratings
FDM	Fused Deposition Modeling
FEA	Finite Element Analysis
FRCs	Fiber-Reinforced Composites
FTIR	Fourier-Transform Infrared Spectroscopy
GFRP	Glass Fiber-Reinforced Polymer
HM	High-Modulus (carbon fiber)
HS	High-Strength (carbon fiber)
ILSS	Interlaminar Shear Strength
IM	Intermediate-Modulus (carbon fiber)
MDH	Magnesium Hydroxide
PA	Polyamide
PEEK	Polyether Ether Ketone
PEKK	Polyether Ketone Ketone
PHA	Polyhydroxyalkanoates
PLA	Polylactic Acid
PP	Polypropylene
ROV	Remotely Operated Vehicle
RTM	Resin Transfer Molding
S-glass	High-Strength Glass Fiber
SEM	Scanning Electron Microscopy
SHM	Structural Health Monitoring
TCPs	Thermoplastic Composite Pipes
Td	Thermal Decomposition Temperature
Tg	Glass Transition Temperature
TGDDM	Tetraglycidyl Diaminodiphenyl Methane
VARTM	Vacuum-Assisted Resin Transfer Molding
